# Assembly and comparative analysis of the complete mitochondrial genome of *Isopyrum anemonoides* (Ranunculaceae)

**DOI:** 10.1371/journal.pone.0286628

**Published:** 2023-10-05

**Authors:** Gulbar Yisilam, Zhiyou Liu, Rayhangul Turdi, Zhenzhou Chu, Wei Luo, Xinmin Tian

**Affiliations:** 1 Xinjiang Key Laboratory of Biological Resources and Genetic Engineering, College of Life science and Technology, Xinjiang University, Urumqi, Xinjiang, China; 2 City Management and Service Centre of Tiemenguan, Xinjiang, China; 3 Key Laboratory of Ecology of Rare and Endangered Species and Environmental Protection, College of Life Science, Guangxi Normal University, Ministry of Education, Guilin, 541004, China; Agri Biotech Foundation and Retired Professor, University of Hyderabad, INDIA

## Abstract

Ranunculaceae is a large family of angiosperms comprising 2500 known species—a few with medicinal and ornamental values. Despite this, only two mitochondrial genomes (mitogenomes) of the family have been released in GenBank. *Isopyrum anemonoides* is a medicinal plant belonging to the family Ranunculaceae, and its chloroplast genome has recently been reported; however, its mitogenome remains unexplored. In this study, we assembled and analyzed the complete mitochondrial genome of *I*. *anemonoides* and performed a comparative analysis against different Ranunculaceae species, reconstructing the phylogenetic framework of *Isopyrum*. The circular mitogenome of *I*. *anemonoides* has a length of 206,722 bp, with a nucleotide composition of A (26.4%), T (26.4%), C (23.6%), and G (23.6%), and contains 62 genes, comprising 37 protein-coding genes (PCGs), 22 transfer RNA (tRNA) genes, and three ribosomal RNA (rRNA) genes. Abundantly interspersed repetitive and simple sequence repeat (SSR) loci were detected in the *I*. *anemonoides* mitogenome, with tetranucleotide repeats accounting for the highest proportion of SSRs. By detecting gene migration, we observed gene exchange between the chloroplast and mitogenome in *I*. *anemonoides*, including six intact tRNA genes, six PCG fragments, and fragments from two rRNA genes. Comparative mitogenome analysis of three Ranunculaceae species indicated that the PCG contents were conserved and the GC contents were similar. Selective pressure analysis revealed that only two genes (*nad1* and *rpl5*) were under positive selection during their evolution in Ranunculales, and two specific RNA editing sites (*atp6* and *mttB*) were detected in the *I*. *anemonoides* mitogenome. Moreover, a phylogenetic analysis based on the mitogenomes of *I*. *anemonoides* and the other 15 taxa accurately reflected the evolutionary and taxonomic status of *I*. *anemonoides*. Overall, this study provides new insights into the genetics, systematics, and evolution of mitochondrial evolution in Ranunculaceae, particularly *I*. *anemonoides*.

## Introduction

*Isopyrum* belongs to the family Ranunculaceae. The genus contains four species, mainly distributed in the temperate regions of the Northern Hemisphere [1]. Two species, *I*. *manshuricum* and *I*. *anemonoides*, are found in China [2,3]. *Isopyrum* plants contain various chemical components, including benzylisoquinoline alkaloids, flavonoids, triterpenoid saponins, cyanides, and lactones, and most of its genera are used in traditional Chinese medicine to clear away heat and detoxification, relieve swelling and pain, and treat cardiovascular diseases [4]. For example, the root uber of *I*. *anemonoides* is highly valued for its medicinal properties [5]. While the chloroplast genome of *I*. *anemonoides* has been recently reported, its mitochondrial genome (mitogenome) remains unexplored.

The mitochondrion is a semi-autonomous organelle with its own genetic material and genetic system, providing a source of chemical energy for eukaryotes through oxidative phosphorylation [6]. The typical angiosperm mitogenome is a double stranded DNA molecule, and its size is extremely variable between different species, ranging from 66 kb (*Viscum scurruloideum*) to over 11.3 mb (*Silene conica*), even among closely related species [7,8]. Angiosperm mitochondrial genomes are known for their low mutation rate, relatively high incidence of RNA editing and trans-splicing of coding sequences, and dynamic structure [9]. Most size and structural variations in angiosperm mitogenomes are related to the uptake of foreign sequences because the mitochondrial genes are obtained from gene transfer between nuclear and plastid genomes or even different species [10,11]. Consequently, the assembly of mitogenomes can be more complex and challenging than that of chloroplast genomes [12]. With the development of next generation sequencing technology, obtaining mitogenome sequences have become more feasible [13–16]. Several studies have indicated that mitogenomes are popular genetic markers for population genetics, molecular ecology, plant classification, and evolution [17,18], such as in *Ginkgo* and *Welwitschia* [19], Oleaceae [20], Dioscoreales [21], and Vitaceae [22]. Despite comprising 2500 known species, data on the mitochondrial genomes of the Ranunculaceae family are lacking, with only two mitochondrial genomes released in GenBank as of December 2022. This limitation hinders the overall understanding of the phylogenetics and adaptive evolution of Ranunculaceae species [23,24].

Given the importance of the plant mitochondrial genome in understanding the phylogenetics and adaptive evolution of Ranunculaceae species, this study aims to sequence and analyze the complete mitogenome of *I*. *anemonoides* and compare it with the mitogenomes of two other Ranunculaceae species, *Aconitum kusnezoffii* (NC053920) [23], and *Anemone maxima* (MT568500) [24]. This study will contribute to exploring the structure and evolution of mitogenomes, and highlight the need for more mitogenomes to be sequenced in the family Ranunculaceae.

## Materials and methods

### Plant materials, DNA sequencing and assembly

Fresh leaves of *I*. *anemonoides* were collected from Jimusaer County in Xinjiang Autonomous Region, China (89˚ 18’ N, 44˚ 00’ E). The plant material was identified by Xinmin Tian (http://sky.xju.edu.cn/info/1151/1956.htm) using the detailed species information in Flora of China (http://www.iplant.cn/foc). The samples were immediately frozen with silica gel and stored at −20°C. As a non-protected plant, collection of the *I*. *anemonoides* is in accordance with the Laws of China’s National Forests and Grasslands Administration (no sampling license is required). Voucher specimens (TXM202006) were deposited at the Herbarium of Xinjiang University (XJU). All methods used in this study were carried out in accordance with relevant guidelines and regulations.

The total genomic DNA was extracted using the Plant Genomic DNA Kit (China Tiangen Biotechnology, Beijing, China) and then sequenced using the Illumina Hiseq 2000 platform (Illumina, San Diego, CA, USA). Raw data from paired-end sequencing was filtered to eliminate low-quality sequences, sequences with high “N” ratios, fragments with lengths less than 25 bp, and unknown nucleotides. The clean data produced a total of 6.13 Gb/6.10 Gb. The high-quality clean reads were assembled using GetOrganelle v.1.7.5 with default settings and k-mer values set to 21, 43, 65, 87, and 127 [25]. Contigs were selected using BLAST software (query coverage ≥ 70% and E-value ≤ 1e–10) with the mitogenome of *Aconitum kusnezoffii* (NC053920) as the reference. Geneious Prime 2022.1.1 (https://www.geneious.com) (Biomatters Ltd., Auckland, New Zealand) was used for mitochondrial contig mapping and alignment to verify quality with custom sensitivity (1% maximum for each gap and mismatches allowed). The final mitogenome was obtained using the Geneious mapper and aligner.

### Mitogenome annotation and analysis

The mitogenome annotation was performed using GeSeq (https://chlorobox.mpimp-golm.mpg.de/GenBank2Sequin.html) [26]. The start and stop codons were corrected with Geneious Prime 2022.1.1. The circular map was generated using OGDRAW (http://ogdraw.mpimp-golm.mpg.de/) [27], and the complete mitogenome sequence of *I*. *anemonoides* has been deposited in GenBank (accession number OP161795). Geneious Prime 2022.1.1 was used to calculate the protein-coding genes (PCGs), the contents of GC and AT, and the proportions of A, C, G, and T in the mitogenome of *I*. *anemonoides*. The relative synonymous codon usage (RSCU) values and the amino acid composition of PCGs were calculated using MEGA v.7.0.26 (https://megasoftware.net/) [28].

### Analysis of repeated sequences

The *I*. *anemonoides* mitogenome was analyzed for repeat distribution. REPuter software (https://bibiserv.cebitec.uni-bielefeld.de/reputer) [29] was used to identify the forward, reverse, palindromic, and complementary repeats with maximum computed repeats, minimal repeat size, and hamming distance set to 5000, 30, and 3, respectively. The tandem repeats with >6 bp repeat units were detected using Tandem Repeats Finder v.4.09 [30] (http://tandem.bu.edu/trf/trf.submit.options.html) with default parameters. Simple sequence repeats (SSRs) are DNA fragments composed of short sequence repeats with a length of 1–6 bp. SSRs were analyzed using the Microsatellite identification tool (https://webblast.ipk-gatersleben.de/misa/) [31]. The repeats of 1, 2, 3, 4, 5, and 6 nucleotide SSRs were set as 10, 5, 4, 3, 3, and 3 repeat numbers, respectively.

### Identification of mitochondrial plastid DNAs (MTPTs)

To identify plastid-derived DNA fragments in the mitogenome, we compare the plastome of *I*. *anemonoides* with the mitogenome. The plastid genome data of *I*. *anemonoides* was obtained from our previous study (OM457045) [32]. We used Tbtools v.0.668 (https://www.tbtools.com/) [33] to identify sequences shared between the plastid and mitogenome with screening criteria set to a matching rate of ≥ 70%, E-value of ≤ 1e-5, and length of ≥ 40.

### Selective pressure analysis and prediction of RNA editing sites

We calculated the non-synonymous substitution rate (d*N*) and synonymous substitution rate (d*S*) of each PCG in *I*. *anemonoides*, *A*. *kusnezoffii*, and *A*. *maxima* of the Ranunculaceae family. First, the PCGs of these species were extracted using PhyloSuite v.1.2.2 [34] (http://phylosuite.jushengwu.com/) and separately aligned using MAFFT v.7.407 [35], with all stop codons removed. The alignments were manually checked for accuracy. We then used the yn00 module in PAML v.4.9 [36] to calculate the d*N* and d*S* values, estimating pairwise nucleotide substitution rates with the following parameters: verbose = 0; icode = 0; weighting = 0; commonf3 × 4 = 0 (use one set of codon freqs. for all pairs); ndata = 1d*N*/d*S*. For RNA editing site analysis, all mitogenome PCGs of the three species were extracted using the same approaches as above, and RNA editing sites were predicted using PREP-Mt (http://prep.unl.edu/) [37], with a cutoff value of 0.2.

### Phylogenetic analysis

#### Phylogenetic analysis

To verify the placement of our newly sequenced *I*. *anemonoides* mitogenome within the Ranunculaceae family, we downloaded 15 complete mitogenome sequences from the National Center for Biotechnology Information (NCBI) database to construct a phylogenetic tree (S1 Table). *Ginkgo biloba* (KM672373) was used as an outgroup. The 25 mitochondrial PCGs (*atp1*, *atp4*, *atp6*, *atp8*, *atp9*, *ccmB*, *ccmC*, *ccmFc*, *ccmFn*, *cob*, *cox1*, *cox3*, *matR*, *nad1*, *nad2*, *nad3*, *nad4*, *nad4L*, *nad5*, *nad6*, *nad7*, *nad9*, *rps12*, *rps3*, *and rps4*) were extracted using PhyloSuite v.1.2.2 [34] and aligned using MAFFT v.7.407 [35] in PhyloSuite. The aligned nucleotide sequences were concatenated to construct Maximum-Likelihood (ML) and Bayesian inference (BI) phylogenies. We used IQ-TREE [38] for ML analysis with the automatic and FreeRate heterogeneity options under optimal evolutionary models. Branch support values were calculated using the ultrafast bootstrap and the SH-aLRT branch test approximation with 1,000 replicates. For BI analysis, we used MrBayes v.3.2.6 [39] with ModelFinder to select the best-fit model using the Akaike information criterion (AIC), and the BI phylogeny was generated with a total chain length of 2,000,000 (burn-in of 100,000 trees) and sampling every 100 cycles under the K2P+G4 substitution model. The final phylogenetic topologies were visualized using Figtree v1.4.4 (http://tree.bio.ed.ac.uk/software/figtree/).

## Results

### Features of the *I*. *anemonoides* mitogenome

**Fig 1:**
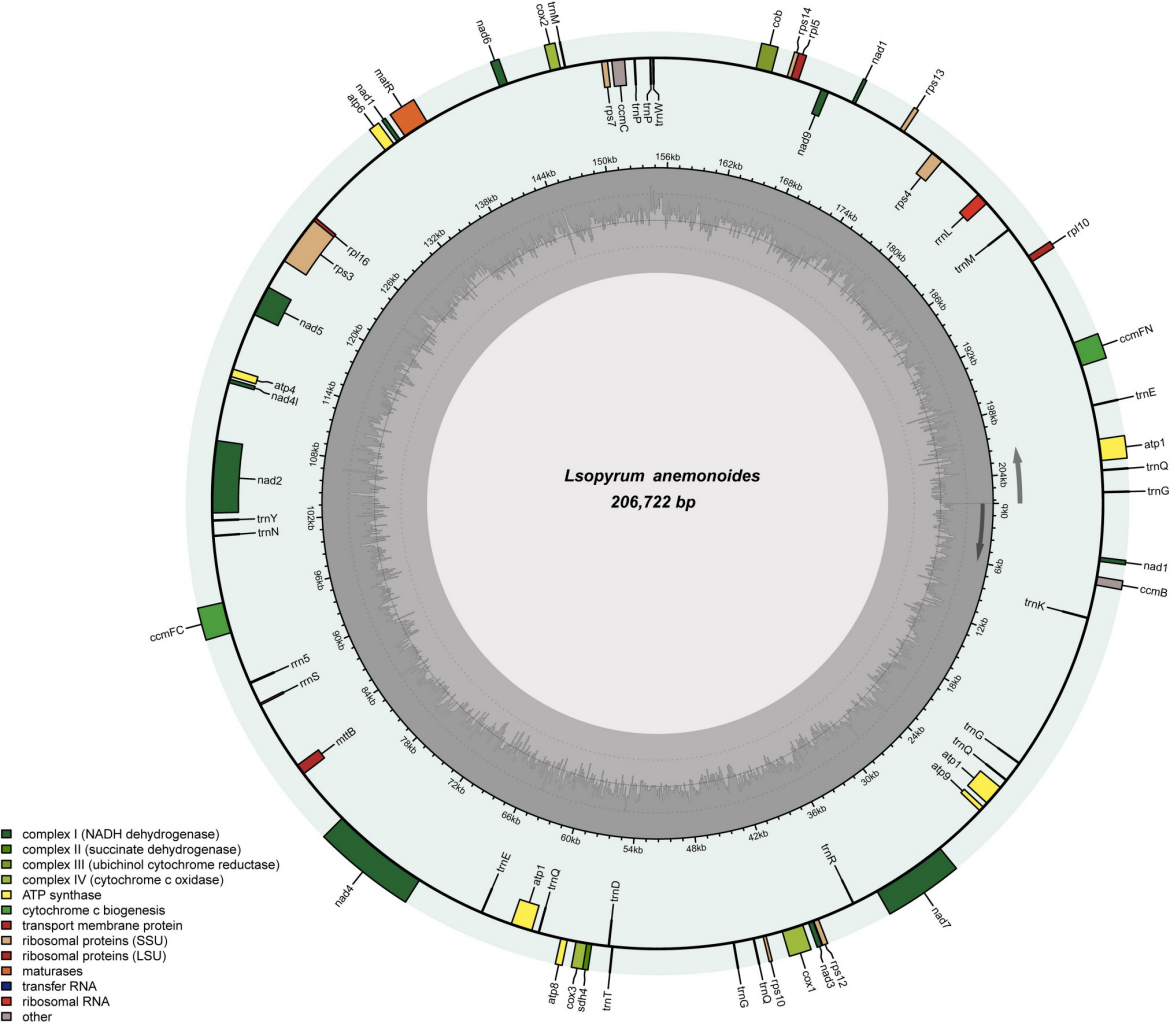
Genome map of *Isopyrum anemonoides* mitogenomes. Different colors represent different genes. The inner genes are transcribed clockwise, and the outer genes are transcribed counterclockwise. The innermost gray pattern represents the GC content.

The complete circular mitogenome of *I*. *anemonoides* was 206,722 bp in length, with the typical structure observed in most land plants (Fig 1). The nucleotide composition of the mitogenome was determined to be 26.4% A, 26.4% T, 23.6% G, and 23.6% C, resulting in a GC content of 47.2% and an AT content of 52.8%. A total of 61 genes were identified in the mitogenome of *I*. *anemonoides*, comprising 37 PCGs, three ribosomal RNA (rRNA) genes, and 21 transfer RNA (tRNA) genes, with 20 exons and 12 introns (Table 1). The PCGs were classified into nine categories: NADH dehydrogenases (nine genes), succinate dehydrogenases (one gene), ubiquinol cytochrome c reductases (one gene), cytochrome c oxidases (three genes), ATP synthases (five genes), ribosomal proteins (10 genes), maturases (one gene), transport membrane proteins (one gene), and cytochrome c biogenesis (four genes) (S2 Table).

**Table 1:** General features of mitogenomes from three Ranunculaceae species.

	*Isopyrum anemonoides*	*Anemone maxima*	*Aconitum kusnezoffii*
Accession	OP161795	MT568500	NC053920
Size (bp)	206,722	1,122,546	440,720
Genes	61	62	69
Size (bp)	53,827	78,312	210,980
Protein-coding	37	39	37
Size (bp)	51,185	71,626	207,073
tRNAs	21	18	29
tRNA length (bp)	1,497	1,324	2,131
rRNA genes	3	3	3
Size (bp)	1,149	5,362	1,847
A content	26.4%	27.0%	26.5%
T content	26.4%	26.8%	26.6%
C content	23.6%	23.2%	23.4%
G content	23.6%	23.0%	23.5%
C + G content	47.2%	46.2%	46.9%

The 37 PCGs varied from 222 bp (rps10) to 7,322 bp (nad4), with a total length of 51,185 bp, accounting for 24.76% of the *I*. *anemonoides* mitogenome. The lengths of tRNA and rRNA were 1,497 bp and 1,149 bp, making up 0.72% and 0.56% of the mitogenome, respectively (Table 1). Among the seven intron-containing genes in the *I*. *anemonoides* mitogenome, *nad7* has three introns, whereas *rps3*, *nad5*, and *ccmFC* have one intron each. In addition, *nad2*, *nad4*, and *nad1* have two introns each. The introns in the *nad1* gene have lengths of 40,561 bp and 35,117 bp (S2 Table).

### Codon usage analysis of PCGs

**Fig 2:**
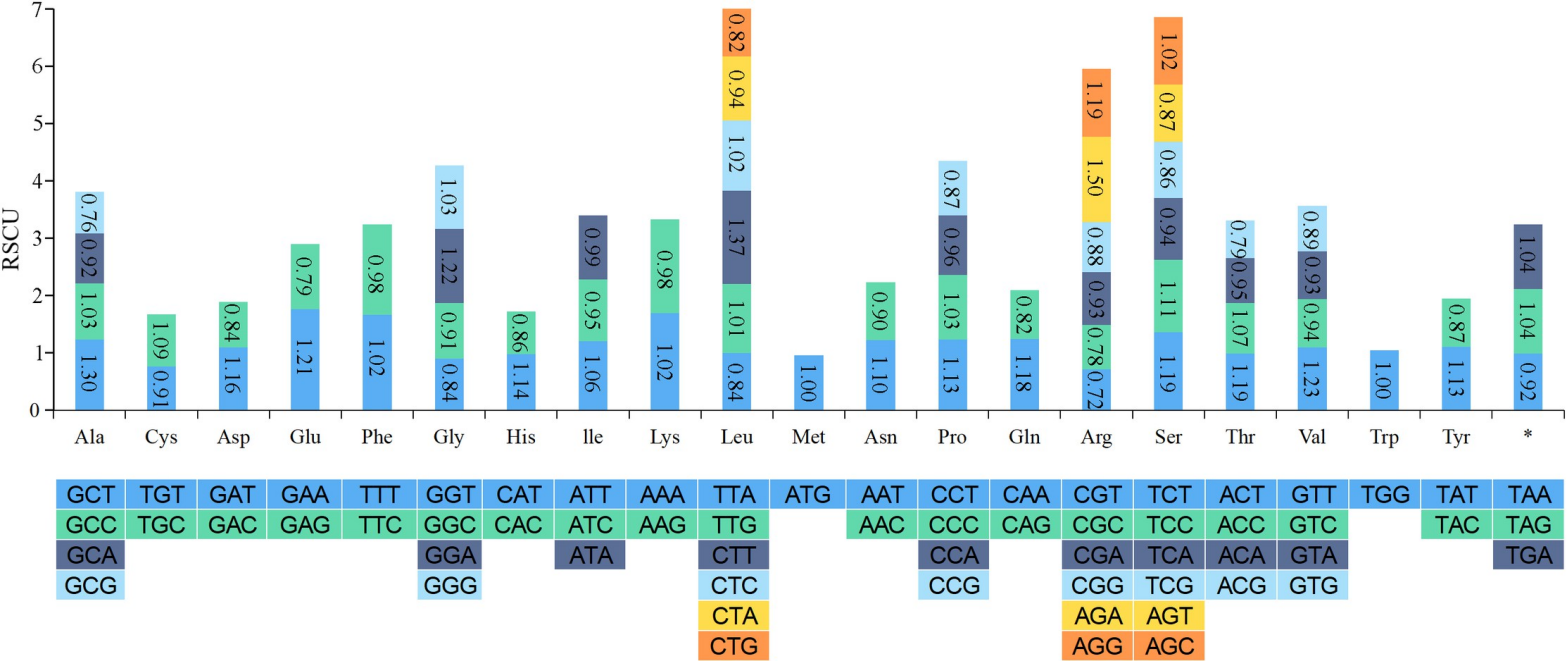
Relative synonymous codon usage (RSCU) in the *Isopyrum anemonoides* mitogenome. Codon families are shown on the *x*-axis. The RSCU values represent the frequency of each codon usage relative to the expected frequency based on uniform synonymous codon usage.

Most PCGs in the *I*. *anemonoides* mitogenome had the typical ATG start codon, except for *atp6* and *mttB*, which had TTG and CTG as their start codons, respectively (S2 Table). Leucine (Leu) was the most frequently used amino acid. At the same time, methionine (Met) and tryptophan (Trp) were the least used amino acids among the 37 PCGs. We calculated the RSCU of the 37 PCGs in the *I*. *anemonoides* mitogenome and observed that the RSCU values of 31 codons were greater than 1.00 (Fig 2). These results indicate a strong bias toward a high representation of NNA and NNT, similar to other land plant species.

### Repeat sequence analysis

**Fig 3:**
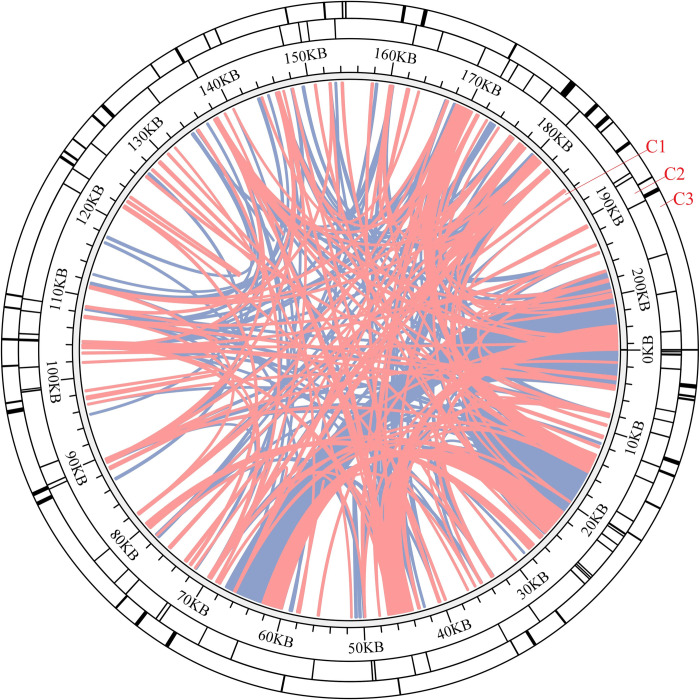
Repeat analysis of the *I*. *anemonoides* mitogenome. The C1 circle shows the dispersed repeats connected with blue and pink arcs from the center going outward: The C2 circle shows the tandem repeats as short bars. The C3 circle shows the microsatellite sequences identified using MISA.

Repeat sequence analysis of the *I*. *anemonoides* mitogenome revealed a total of 507 interspersed repetitive sequences (>30 bp), including 261 palindromic (67,304 bp) and 246 forward (47,398 bp); no complementary or reverse repeats were observed (Fig 3). The total length of the dispersed repetitive sequences was 114,702 bp, which accounted for 55.49% of the *I*. *anemonoides* mitogenome. These repeats ranged from 31 to 1,624 bp (nine were longer than 1 kb) (S3 Table). Most of these dispersed repeats were observed in the *nad1* introns and gene spacer regions, and a few were located in the gene coding region. Additionally, 52 tandem repeats were detected, with repeat lengths ranging from 27 to 305 bp (Fig 3 and S4 Table). Most of these tandem repeats were observed in two copies, with 21% having a length of 0–50 bp, 29% with a length of 51–100 bp, and 50% with a length larger than 100 bp (S4 Table). Additionally, a total of 68 SSRs (805 bp) were detected in the mitogenome (S5 Table), comprising 19 (28.0%) mono-, 10 (14.70%) di-, 5 (7.35%) tri-, 31 (45.60%) tetra-, and 3 (4.41%) pentanucleotide repeats.

### Identification of MTPTs

In this study, we observed 15 DNA fragments shared between the chloroplast and mitogenomes with a total length of 7,302 bp, accounting for 3.53% of the mitogenome (Table 2). These fragment lengths ranged from 59 to 1,576 bp, and the sequences exhibited more than 95% similarity in the mitochondrial and chloroplast genomes. Among these shared fragments, six were intact tRNA genes, six were partial PCGs, and the remaining fragments were part of the chloroplast ribosome RNA, namely *trnW*-*CCA*, *trnP*-*UGG*, *trnD*-GUC, *trnN-GUU*, *trnM-CAU*, *trnT-GGU* and *psbB*, *petL*, *petG*, *rps14*, *psbD*, *ndhF*, *rrn16*, and *rrn23* (Table 2).

**Table 2:** Fragments transferred from chloroplast to mitochondria in *Isopyrum anemonoides*.

Alignment length	Identity%	Mismatches	Gap opens	cp start	cp end	mt start	mt end	Gene
1,576	100	0	0	73,089	74,664	157,752	156,177	*psbB*
995	97.487	2	1	66,045	67,039	155,604	154,633	*petL* *petG* *trnW*-*CCA*
921	95.114	36	6	140,154	141,071	88,163	87,249	*rrn16*
793	99.369	1	4	143,780	144,568	172,777	173,569	
473	100	0	0	37,992	38,464	133,255	132,783	*rps14*
441	100	0	0	34,206	34,646	5,320	4,880	*psbD*
431	100	0	0	67,078	67,508	154,638	154,208	*trnP*-*UGG*
436	82.569	53	14	135,303	135,728	183,777	183,355	*rrn23*
356	98.596	5	0	134,648	135,003	184,416	184,061	*rrn23*
354	97.74	1	3	30,747	31,094	55,521	55,169	*trnD*-*GUC*
193	99.482	1	0	116,140	116,332	174,949	175,141	*ndhF*
113	94.69	6	0	144,282	144,394	25,456	25,344	
84	96.429	2	1	112,791	112,873	100,992	100,909	*trnN*-*GUU*
77	90.909	7	0	53,202	53,278	148,205	148,129	*trnM*-*CAU*
59	94.915	3	0	32,279	32,337	55,043	54,985	*trnT*-*GGU*

### Comparison of mitogenome sizes and GC contents between *I*. *anemonoides* and the other two species from Ranunculaceae

**Fig 4:**
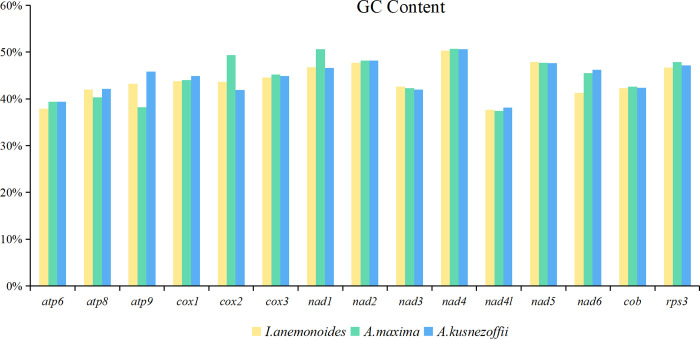
The GC contents of 3 three Ranunculaceae species mitogenome. Comparison of GC contents of 33 PCGs between *Isopyrum anemonoides*, *Anemone maxima*, and *Aconitum kusnezoffii*.

We compared the genome sizes, GC contents, PCGs, rRNAs, and tRNAs of *I*. *anemonoides* with those of other published Ranunculaceae mitogenomes, including *Anemone maxima* and *Aconitum kusnezoffii* (Table 1). Their GC content was similar, ranging from 46.2% to 47.2% (Fig 4). However, their mitogenome sizes varied greatly, with *A*. *maxima* having the largest mitogenome at 1122,546 bp, followed by *A*. *kusnezoffii* at 440,720 bp and *I*. *anemonoides* at 206,722 bp. These three plants have similar proportions of rRNA genes. However, the proportions of tRNA and PCGs are significantly different among families (Table 1). This variation may be attributed to differences in mitogenome sizes.

### Gene duplications and losses in the mitogenomes of the three Ranunculaceae species

Mitogenome loss is a common phenomenon in land plants; therefore, the fate of mitogenome loss has received increasing attention. However, certain genes, such as the Complex I gene (*nadX*), Complex III gene (*cob*), Complex IV gene (*cox1-3*), Complex V gene, and *ccmX* gene, are relatively conserved. In contrast, other types of genes may be lost in different plants (Table 3). Here, we compared the PCGs of *I*. *anemonoides*, *A*. *maxima*, and *A*. *kusnezoffii*. These three species have 37, 39, and 37 PCGs, respectively. As shown in Table 3, PCG loss events occurred in the mitogenomes of these species. For example, the *atp1* gene was duplicated three times in *I*. *anemonoide*s. In addition, the sdh3 and rps1 genes were lost compared to the other two species. *A*. *maxima* has an almost complete mitogenome, with only two genes (*rps10* and *rps14*) lost. In contrast, the *rps2*, *rps11*, *rps19*, and *rpl2* genes were lost from the *A*. *kusnezoffii* and *I*. *anemonoide*s mitogenomes. In addition, the number of rRNA genes in the three species was the same; however, the number and type of tRNA genes differed.

**Table 3:** Gene duplication and loss in mitogenomes of the three Ranunculaceae species.

Product group	*anemonoides*	*maxima*	*A*. *kusnezoffii*
Complex I (NADH dehydrogenase)	*nad1*, *nad2*, *nad3*, *nad4*, *nad4L*, *nad5*, *nad6*, *nad7*, *nad9*	*nad1*, *nad2*, *nad3*, *nad4*, *nad4L*, *nad5*, *nad6*, *nad7*, *nad9*	*nad1*, *nad2*, *nad3*, *nad4*, *nad4L*, *nad5*, *nad6*, *nad7*, *nad9*
Complex II (Succinate dehydrogenase)	*sdh4*	*sdh3*, *sdh4*	*sdh3*, *sdh4*
Complex III (Ubiquinol cytochrome c reductase)	*cob*	*cob*	*cob*
Complex IV (Cytochrome c oxidase)	*cox1*, *cox2*, *cox3*	*cox1*, *cox2*, *cox3*	*cox1*, *cox2*, *cox3*
Complex V (ATP synthase)	*atp1*(3), *atp4*, *atp6*, *atp8*, *atp9*	*atp1*(2), *atp4*, *atp6*, *atp8*, *atp9*	*atp1*, *atp4*, *atp6*, *atp8*, *atp9*
Ribosomal proteins (LSU)	*rpl5*, *rpl10*, *rpl16*, *rps3*, *rps4*, *rps7*, *rps12*, *rps13*	*rpl5*, *rpl10*, *rpl16*, *rps3*, *rps4*, *rps7*, *rps12*, *rps13*	*rpl5*, *rpl10*, *rpl16*, *rps3*, *rps4*, *rps7*, *rps12*, *rps13*
Ribosomal proteins (SSU)	*rps14*, *rps10*	*rps1*, *rpl2*, *rps2*, *rps11*, *rps19*	*rps14*, *rps10*, *rps1*
Maturases	*matR*	*matR*	*matR*
Transport membrane protein	*mttB*	*mttB*	*mttB*
Cytochrome c biogenesis	*ccmB*, *ccmC*, *ccmFn*, *ccmFc*	*ccmB*, *ccmC*, *ccmFn*, *ccmFc*	*ccmB*, *ccmC*, *ccmFn*, *ccmFc*
Ribosomal RNAs	*rrn5*, *rrnL*, *rrnS*	*rrn5*, *rrn18*, *rrn26*	*rrn5*, *rrnL*, *rrnS*
Transfer RNAs	*trnD-GUC*	*trnC(GCA)*(2)	*trnD-GUC*
	*trnE-UUC*(2)	*trnD(GUC)*(2)	*trnE-UUC*(2)
	*trnG-GCC*(3)	*trnfM(CAU)*	*trnF-GAA*(2)
	*trnK-UUU*	*trnG(GCC)*	*trnG-GCC*(3)
	*trnM-CAU*	*trnH(GUG)*(2)	*trnK-CUU*(2)
	*trnN-GUU*	*trnI(CAU)*	*trnM-CAU*(4)
	*trnP-UGG*(3)	*trnK(UUU)*(2)	*trnN-GUU*(2)
	*trnQ-UUG*(4)	*trnM(CAU)*	*trnP-UGG*(4)
	*trnR-UCG*	*trnN(GUU)*	*trnQ-UUG*(4)
	*trnT-GGU*	*trnP(UGG)(2)*	*trnR-UCG*
	*trnW-CAC*	*trnQ(UUG)*	*trnT-GGU*
	*trnW-CCA*	*trnW(CCA)*	*trnW-CCA*
	*trnY-GUA*	*trnY(GUA)*	*trnY-GUA*

### Prediction of RNA editing sites in PCGs

**Fig 5:**
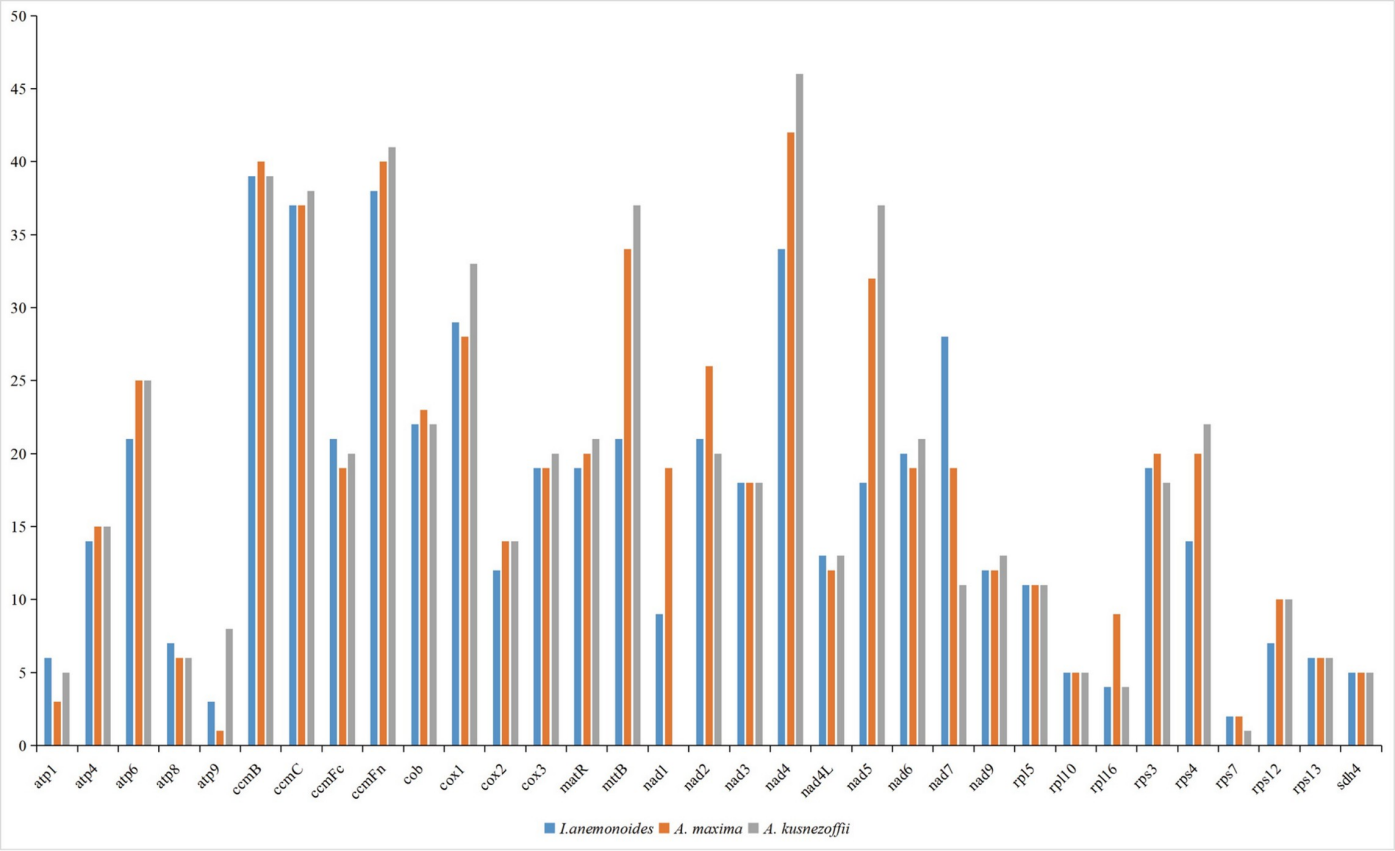
The distribution of RNA editing sites in three Ranunculaceae species mitogenome.

RNA editing is a process that occurs in the mitochondria and plastids of plants. In plant mitogenomes, RNA editing changes the genomic information by converting cytosine (C) to uridine (U). In this study, we predicted the RNA editing sites of 33 common PCGs in the mitogenomes of three Ranunculaceae species. Our results showed that the number of RNA editing sites predicted in the *I*. *anemonoides*, *A*. *maxima*, and *A*. *kusnezoffii* mitogenomes was 554, 611, and 605, respectively. Furthermore, all editing sites in the three species occurred only at the first and second positions of the triplet codes, with none observed at the third position. Among the 33 PCGs, the *ccmFn*, *ccmB*, *ccmC*, and *nad4* genes had the most editing sites (Fig 5), whereas few RNA editing sites were observed in *rpl10*, *rps7*, and *sdh4*.

### Substitution rates of protein-coding genes

**Fig 6:**
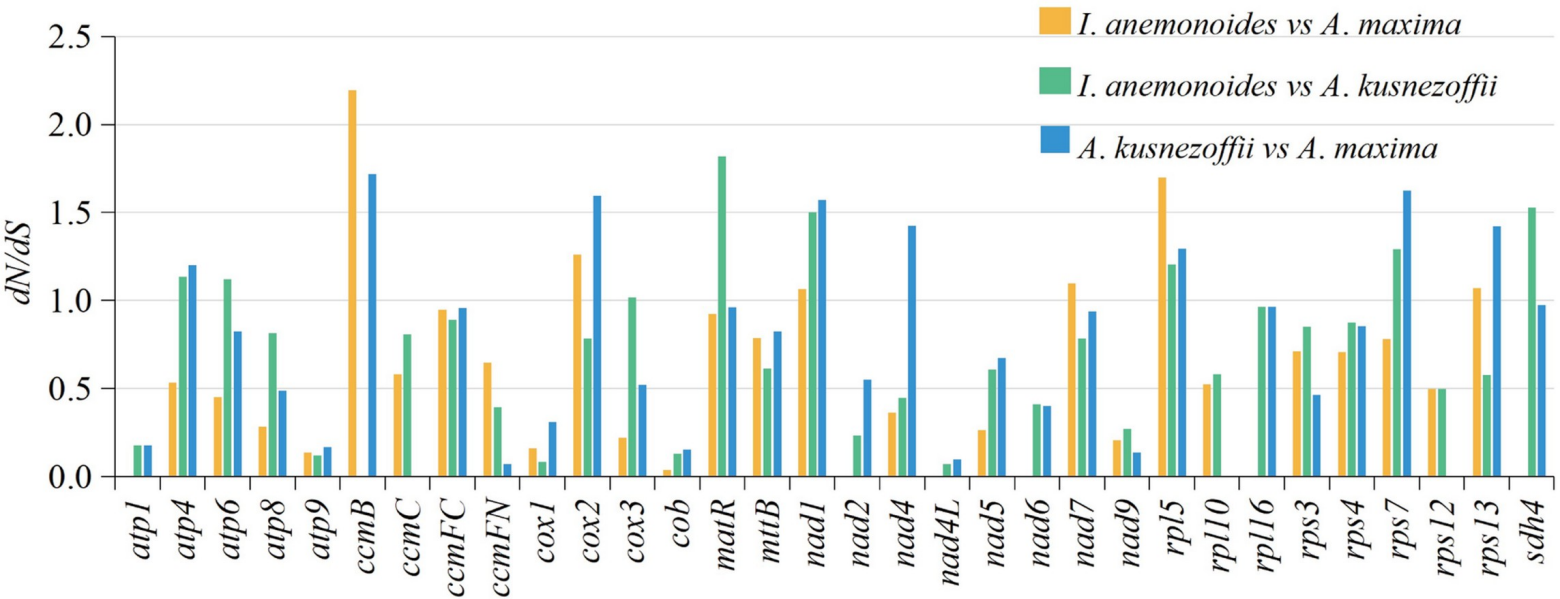
d*N*/d*S* ratios of 32 PCGs in *Isopyrum anemonoides*, *Anemone maxima*, *and Aconitum kusnezoffii*.

The pairwise d*N*/d*S* ratio can be used to determine the presence of selective pressure on specific PCGs during evolution. In general, d*N*/d*S* = 1 represents neutral selection, while d*N*/d*S* >1 or d*N*/d*S* <1 represents positive or negative selection, respectively. In this study, the pairwise d*N*/d*S* ratio was determined for 32 PCGs common to the *I*. *anemonoides*, *A*. *maxima*, and *A*. *kusnezoffii* mitogenomes. The *nad3* gene was conserved in all three species and was thus excluded from this analysis. The results showed that most d*N*/d*S* ratios were less than 1.0, mainly for *atp1*, *atp9*, *nad2*, *nad4L*, *nad6*, *rpl16*, *rps12*, and *cob*, suggesting that most of the PCGs were subject to negative selection during their evolution (Fig 6 and S6 Table). In contrast, the d*N*/d*S* ratios of *ccmB*, *cox2*, *nad1*, *rpl5*, and *rps1* were greater than 1.0 in the *I*. *anemonoides* and *A*. *kusnezoffii* mitogenomes. In addition, the d*N*/d*S* ratios of *atp4*, *atp6*, *cox2*, *cox3*, *matR*, *nad1*, *rpl5*, *rps7*, and *sdh4* genes were greater than 1.0 in the *I*. *anemonoides* and *A*. *maxima* mitogenomes (Fig 6). Only two genes, *nad1* and *rpl5*, had d*N*/d*S* ratios greater than 1.0 in all three species, which indicated these genes were under positive selection during their evolution.

### Phylogenetic analysis

In this study, a phylogenetic analysis was conducted on *I*. *anemonoides* and 15 other species, including 11 dicotyledonous, three monocots, and one gymnosperm (designated as outgroups). Maximum likelihood (IQ-TREE) and Bayesian (MrBayes) analyses produced identical topologies, strongly supporting the separation of dicotyledonous from monocots and the separation of angiosperms from gymnosperms with a high support value. *I*. *anemonoides*, *A*. *kusnezoffii*, and *A*. *maxima* formed a closely-clustered branch with high confidence (100% SH-aLRT support, 100% ultrafast bootstrap support in ML, posterior probability 100% in BI). These findings provide valuable insights into the evolutionary history of *I*. *anemonoides* and serve as a foundation for further research.

## Discussion

The mitochondrion is a semi-autonomous organelle that contains its own genetic material and genetic system, and it provides most of the energy for eukaryotes [40]. Mitogenomes in higher plants vary significantly in size and complexity within families and genera. For example, *Cucumis melo* (2.9 Mt) and *Citrullus lanatus* (379,236 nt) [41], and *Silene conica* (11,318,806 nt) and *S*. *latifolia* (253,413 nt) [42,43]. Due to this size variation and their complex structure, which includes frequent intra-genomic rearrangement, repeat sequences, gene transfer/loss, and highly variable levels of RNA editing, plant mitogenomes are more complex than those of animals [44,45]. In this study, we sequenced and annotated the mitogenome of *I*. *anemonoides* and conducted a comparative analysis with two species from the Ranunculaceae family, *A*. *maxima* and *A*. *kusnezoffii*. The GC content of the three species was similar, ranging from 46.2% to 47.2%. However, the size of the mitogenomes varied greatly (Table 1). PCGs account for 24.76% of the *I*. *anemonoides* mitogenome, with the *atp1* gene having three copies. PCGs play important roles in the plant mitogenome. For example, the mitochondrial *atp1* gene, which encodes the protein ATP1 (α-subunit of mitochondrial ATP synthase F_1_), is expressed differentially between sterile male lines in wheat [46]. The gene *cox1* is involved in the fertility transformation of the thermosensitive male-sterile line YS3038 in wheat [47]. Moreover, similar to other angiosperms, most PCGs in the *I*. *anemonoides* mitogenome use the typical ATG start codon [48,49], whereas *atp6* and *mttB* genes use TTG and CTG as start codons, respectively, which are modified by RNA editing.

Repeat sequences are fragments that occur at multiple locations in the genome and can contain much genetic information, which is useful for developing markers for population and evolutionary analyses [50–52]. In this study, we confirmed the presence of interspersed, tandem, and SSR repeats in the *I*. *anemonoides* mitogenome. Among them, dispersed repetitive sequences were the most prevalent, accounting for 55.49% (114,702 bp) of the *I*. *anemonoides* mitogenome (Fig 3). However, the repeat types found in the *I*. *anemonoides* mitogenome are different from those found in higher plants, such as *Mangifera persiciformis* (750,898 bp), *M*. *longipes* (728,635 bp), and *M*. *sylvatica* (714,426 bp) [53]. Differences in the size of plant mitogenomes can be explained by variations in the size and type of repeat sequences present in mitochondrial genomes [54].

In plant genomes, gene transfer from chloroplasts to mitogenomes is common [55]. Previous research has shown that gene transfer from organelle genomes to the nuclear genome is the most common direction in angiosperms, followed by transfers from the nuclear and plastic genomes to the mitogenome. In this study, we observed 15 gene fragments that have been transferred from the chloroplast genome to the mitogenome (Table 2). These sequences have a total length of 7,302 bp, accounting for 3.53% of the mitogenome. Among these, six complete tRNA genes were transferred, suggesting that they are more conserved than the PCGs and play an important role in the mitogenome [56,57]. The transfer of genes from the chloroplast to the mitogenome may contribute to the high degree of rearrangements observed among mitochondrial genomes and promote genetic diversity, thus impacting eukaryotic evolution [58,59].

Additionally, we investigated gene loss events in the mitogenomes of *I*. *anemonoides*, *A*. *maxima*, and *A*. *kusnezoffii* (Table 3). Compared to higher plants, the mitochondrial genomes of these species exhibit relatively conserved genes, such as Complex I, Complex III–Complex V, rRNAs, transport membrane proteins, and maturases. Among the three species, *A*. *maxima*, has a considerably complete mitogenome, with only two genes (*rps10* and *rps14*) lost. In contrast, *rps2*, *rps11*, *rps19*, and *rpl2* genes were lost from the *A*. *kusnezoffii* and *I*. *anemonoides* mitogenomes. Moreover, the numbers and types of tRNA genes differed. Mitogenomes contain a large number of non-coding sequences and repeat sequences, and during evolution, gene substitution and functional transfer occur, leading to a considerable loss and transfer of genes. Typically, the missing functional genes in plant mitogenomes are compensated by the nuclear genome [60]. Furthermore, tRNAs in plant mitogenomes are frequently lost and replaced by chloroplast-origin tRNAs, which are eventually transported or transferred to the mitochondrial genome [41]. Because of the rapid evolution of mitogenomes and the loss of functional genes, gene loss can vary significantly between species, even among those belonging to the same family or genus.

RNA editing is a process that modifies genetic information at the transcriptional RNA level, occurring in the chloroplast and mitogenomes of higher plants and contributing to protein folding [61]. Many studies have shown that RNA editing of the mitogenome is closely related to certain cultivated plant traits, such as those in *Sorghum bicolor* [62,63]. In this study, we estimated approximately 554 RNA editing sites in *I*. *anemonoides*, 611 RNA editing sites in *A*. *maxima*, and 605 RNA editing sites in *A*. *kusnezoffii* mitogenomes. Among the 33 PCGs, the *ccmFn*, *ccmB*, *ccmC*, and *nad4* genes had the most editing sites (Fig 5). In contrast, few RNA editing sites were found in *rpl10*, *rps7*, and *sdh4*. Ribosomal protein genes have fewer RNA editing sites, while *ccmB* and *ccmFN* genes have more editing sites; this has also been observed in other plants, such as *Brassica napus* [64] and *Oryza sativa* [65]. RNA editing can create start and/or stop codons, resulting in more conserved proteins with higher homology than other proteins, allowing for better expression of mitochondrial genes. For example, in this study, RNA editing modified the start codons of the *atp6* and *mttB* genes to TTG and CTG, respectively. Other studies have also reported these events, such as in *Acer truncatum* [59] and *Lycopersicon esculentum* [66].

**Fig 7:**
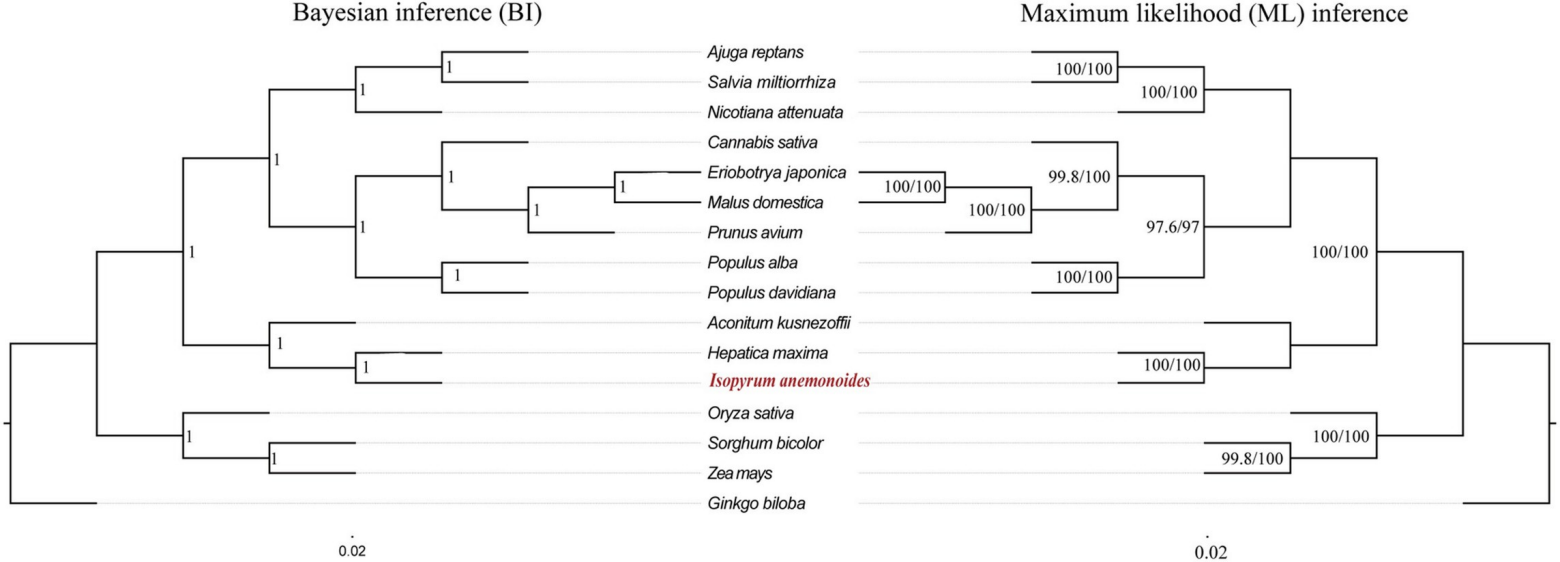
Maximum likelihood and Bayesianconsensus tree of *I*. *anemonoides* with other 15 plant species. ML and BI consensus trees were constructed based on the sequences of 25 conserved protein-coding genes using IQ-TREE (ML) and MrBayes (BI). The species and GenBank accession numbers used in the phylogenetic analysis are listed in Additional File 1.

Furthermore, most mitogenomes are highly conserved and have undergone neutral and negative selections [55]. The d*N*/d*S* analysis results showed that most of the *I*. *anemonoides* PCGs were under negative selection, indicating that the PCGs in the mitogenome are conserved across land plants [56]. Similar negative selection patterns were observed in another herb, *S*. *glauca* [56]. However, comparative analysis results indicated that the d*N*/d*S* values of two genes (*nad1*, and *rpl5*) were greater than 1.0 in all three species, suggesting that these genes were under positive selection during evolution (Fig 6). *nad1* is one of the subunits of ATP synthase and is an important mitogenome in plant breeding. Studies have shown that cytoplasmic male sterility is closely related to the function of *nad1* [67]. Meanwhile, *rpl5* is a ribosomal protein gene involved in gene transcriptional regulation [68]. These two genes might have developed novel functions for stress resistance in Ranunculaceae plants under positive selective pressure. Finally, we analyzed the phylogenetic relationship between *I*. *anemonoides* and representative taxa based on mitochondrial genes. Phylogenetic tree analysis showed a clear taxonomic relationship among the taxa. *I*. *anemonoides*, *A*. *kusnezoffii*, and *A*. *maxima* formed one clade with high support values (Fig 7).

## Conclusion

In this study, we assembled and analyzed the complete mitogenome of *I*. *anemonoides* and performed extensive analyses based on the annotated nucleotide sequences, which is a significant milestone in Isopyreae mitogenome research. The circular genome comprises 61 genes: 37 PCGs, three rRNAs, and 22 tRNAs, and exhibits sequence repeats and MTPT fragment migration. Comparative analyses of genome size and GC content indicated that the gene order tended to be conserved among the three species, with 14 core PCGs. Additionally, d*N*/d*S* analysis based on code substitution showed negative selections in most PCGs across the three Ranunculaceae species, indicating mitogenome conservation during evolution. These results and the availability of the mitogenome of *I*. *anemonoides* provide resources for future studies of Isopyreae and related lineages, which is an important clade of Ranunculaceae for comparative genomics and evolutionary research.

## Supporting information

S1 Table Details regarding the genome sequences used for the phylogenetic analysis. (DOCX)

S2 Table Gene type of the mitogenomes in *Isopyrum anemonoides*. (DOCX)

S3 Table Distribution of repeat loci in the mitogenome of *Isopyrum anemonoides*. (DOCX)

S4 Table Tandem repeats detected in the mitogenomes of *Isopyrum anemonoides*. (DOCX)

S5 Table SSRs in the mitochondrial genome of *Isopyrum anemonoides*. (DOCX)

S6 Table Pairwise d*N*/d*S* ratios in different mitochondrial genes of 3 Ranunculaceae plants. (DOCX)
