# Supplementary material for: Assembly and comparative analysis of the complete mitochondrial genome of Isopyrum anemonoides (Ranunculaceae)
Source: PLoS One. 2023 Oct 5;18(10):e0286628. doi: 10.1371/journal.pone.0286628 (PMC10553351; doi:10.1371/journal.pone.0286628)
Supplement: S1 Table — (DOCX) [file pone.0286628.s001.docx]

**S1 Table. species and GenBank accession number used in the phylogenetic analysis**

| species name | mitogenomes |
| --- | --- |
| *Populus davidiana* | NC035157 |
| *Populus alba* | NC041085 |
| *Cannabis sativa* | NCo29855 |
| *Malus domestica* | NCO18554 |
| *Eriobotrya japonica* | NC045228 |
| *Prunus avium* | NCO44768 |
| *Anemone maxima* | MT568500 |
| *Aconitum kusnezoffii* | MW013323 |
| *Nicotiana attenuata* | NCO36467 |
| *Salvia miltiorrhiza* | NC023209 |
| *Ajuga reptans* | NC023103 |
| *Oryza sativa* | JF281153 |
| *Sorghum bicolor* | DQ984518 |
| *Zea mays* | DQ645539 |
| *Ginkgo biloba* | *KM672373* |
