# Supplementary material for: Assembly and comparative analysis of the complete mitochondrial genome of Isopyrum anemonoides (Ranunculaceae)
Source: PLoS One. 2023 Oct 5;18(10):e0286628. doi: 10.1371/journal.pone.0286628 (PMC10553351; doi:10.1371/journal.pone.0286628)
Supplement: S2 Table — (DOCX) [file pone.0286628.s002.docx]

**S2 Table. Gene type of the mitogenomes in *Isopyrum anemonoides***

| **Category** |  | **Name** | **Length** | **Start codon** | **Stop codon** |
| --- | --- | --- | --- | --- | --- |
| **ComplexⅠ (NADH dehydrogenase)** | 9 | *nad1** | 835 | ATG | TAA |
|  |  | *nad2** | 5363 | ATG | TAA |
|  |  | *nad3* | 357 | ATG | TAA |
|  |  | *nad4l* | 273 | ATG | TAA |
|  |  | *nad4** | 7322 | ATG | TAA |
|  |  | *nad5** | 2281 | ATG | TGA |
|  |  | *nad6* | 618 | ATG | TAA |
|  |  | *nad7** | 5631 | ATG | TAG |
|  |  | *nad9* | 573 | ATG | TAA |
| **ComplexⅡ (Succinate dehydrogenase)** | 1 | *sdh4* | 381 | ATG | TGA |
| **ComplexⅢ (Ubiquinol cytochrome c reductase)** | 1 | *cob* | 1182 | ATG | TGA |
| **ComplexⅣ (Cytochrome c oxidase)** |  | *cox1* | 1584 | ATG | TAA |
|  |  | *cox2* | 753 | ATG | TAA |
|  |  | *cox3* | 798 | ATG | TGA |
| **ComplexⅤ (ATP synthase)** | 5 | *atp4* | 573 | ATG | TAA |
|  |  | *atp1*-1 | 1590 | ATG | TAA |
|  |  | *Atp1*-2 | 1527 | ATG | TAG |
|  |  | *Atp1*-3 | 1581 | ATG | TGA |
|  |  | *atp6* | 789 | TTG | TAA |
|  |  | *atp8* | 486 | ATG | TAA |
|  |  | *atp9* | 375 | ATG | TGA |
| **Ribosomal proteins (LSU)** | 3 | *rpl5* | 564 | ATG | TGA |
|  |  | *rpl10* | 507 | ATG | TAG |
|  |  | *rpl16* | 249 | ATG | TAA |
| **Ribosomal proteins (SSU)** | 7 | *rps3** | 3944 | ATG | TAA |
|  |  | *rps4* | 951 | ATG | TAA |
|  |  | *rps7* | 444 | ATG | TAA |
|  |  | *rps10* | 222 | ATG | TAA |
|  |  | *rps12* | 372 | ATG | TAG |
|  |  | *rps13* | 351 | ATG | TGA |
|  |  | *rps14* | 303 | ATG | TAG |
| **Maturases** | 1 | *matR* | 1944 | ATG | TAG |
| **Transport membrane protein** | 1 | *mttB* | 789 | CTG | TAG |
| **Cytochrome c biogenesis** | 4 | *ccmB* | 621 | ATG | TGA |
|  |  | *ccmC* | 954 | ATG | TAG |
|  |  | *ccmFC** | 2304 | ATG | TAA |
|  |  | *ccmFN* | 1794 | ATG | TAG |
| **Ribosomal RNAs** | 3 | *rrn5* | 105 |  |  |
|  |  | *rrnS* | 138 |  |  |
|  |  | *rrnL* | 906 |  |  |
|  | 21 | *trnD-GUC* | 74 |  |  |
|  |  | *trnE-UUC* | 72 |  |  |
|  |  | *trnE*-UUC | 72 |  |  |
|  |  | *trnG*-GCC | 72 |  |  |
|  |  | *trnG*-GCC | 72 |  |  |
|  |  | *trnG*-GCC | 72 |  |  |
|  |  | *trnK*-UUU | 73 |  |  |
|  |  | *trnM-CAU* | 73 |  |  |
|  |  | *trnM*-CAU | 74 |  |  |
|  |  | *trnN*-GUU | 72 |  |  |
|  |  | *trnP*-UGG | 75 |  |  |
|  |  | *trnP-UGG* | 56 |  |  |
|  |  | *trnP-UGG* | 69 |  |  |
|  |  | *trnQ*-UUG | 72 |  |  |
|  |  | *trnQ*-UUG | 72 |  |  |
|  |  | *trnQ*-UUG | 72 |  |  |
|  |  | *trnQ*-UUG | 72 |  |  |
|  |  | *trnR*-UCG | 58 |  |  |
|  |  | *trnT*-GGU | 68 |  |  |
|  |  | *trnW*-CCA | 74 |  |  |
|  |  | *trnY*-GUA | 83 |  |  |
| **Total** | 61 |  |  |  |  |
