# Supplementary material for: Assembly and comparative analysis of the complete mitochondrial genome of Isopyrum anemonoides (Ranunculaceae)
Source: PLoS One. 2023 Oct 5;18(10):e0286628. doi: 10.1371/journal.pone.0286628 (PMC10553351; doi:10.1371/journal.pone.0286628)
Supplement: S3 Table — (DOCX) [file pone.0286628.s003.docx]

**S3 Table. Distribution of repeat loci in the mitogenome of *Isopyrum anemonoides***

| **Size** | **Repeats 1 start** | **Type** | **Size** | **Repeats 2 start** | **E-value** |
| --- | --- | --- | --- | --- | --- |
| **1624** | **1716** | **P** | **1624** | **16462** | **0** |
| **1539** | **22738** | **P** | **1539** | **202039** | **0** |
| **1535** | **22746** | **F** | **1535** | **61092** | **0** |
| **1531** | **61092** | **P** | **1531** | **202039** | **0** |
| **1524** | **22757** | **F** | **1524** | **61103** | **0** |
| **1520** | **61103** | **P** | **1520** | **202039** | **0** |
| **1419** | **64830** | **P** | **1419** | **198228** | **0** |
| **1204** | **3513** | **P** | **1204** | **15258** | **0** |
| **1110** | **66405** | **P** | **1110** | **196863** | **0** |
| **901** | **44038** | **F** | **901** | **203774** | **0** |
| **892** | **21698** | **P** | **892** | **44015** | **0** |
| **869** | **21698** | **P** | **869** | **203774** | **0** |
| **804** | **26750** | **F** | **804** | **79410** | **0** |
| **771** | **0** | **P** | **771** | **19068** | **0** |
| **755** | **13482** | **F** | **755** | **168831** | **0** |
| **740** | **19843** | **P** | **740** | **45953** | **0** |
| **738** | **62777** | **P** | **738** | **201301** | **0** |
| **698** | **63865** | **P** | **698** | **200117** | **0** |
| **694** | **19864** | **P** | **694** | **205835** | **0** |
| **694** | **45978** | **F** | **694** | **205835** | **0** |
| **655** | **929** | **P** | **655** | **18254** | **0** |
| **615** | **14544** | **F** | **615** | **169899** | **0** |
| **608** | **44323** | **P** | **608** | **60177** | **0** |
| **608** | **60177** | **P** | **608** | **204059** | **0** |
| **584** | **21698** | **F** | **584** | **60201** | **0** |
| **574** | **44933** | **F** | **574** | **204668** | **0** |
| **557** | **21141** | **P** | **557** | **204707** | **0** |
| **535** | **21163** | **P** | **535** | **44972** | **0** |
| **414** | **45541** | **F** | **414** | **205317** | **6.71E-240** |
| **400** | **20720** | **P** | **400** | **45511** | **1.8E-231** |
| **370** | **20720** | **P** | **370** | **205317** | **2.08E-213** |
| **350** | **63515** | **P** | **350** | **200813** | **2.28E-201** |
| **299** | **22285** | **F** | **299** | **60785** | **1.04E-167** |
| **299** | **44021** | **P** | **299** | **60785** | **1.04E-167** |
| **282** | **60785** | **P** | **282** | **203774** | **1.68E-157** |
| **270** | **44050** | **P** | **270** | **60785** | **3.34E-153** |
| **219** | **64612** | **P** | **219** | **199849** | **1.69E-122** |
| **200** | **72608** | **F** | **200** | **121846** | **4.65E-111** |
| **171** | **173557** | **P** | **171** | **176421** | **1.34E-93** |
| **157** | **66249** | **F** | **157** | **182659** | **1.7E-82** |
| **150** | **21380** | **F** | **150** | **43004** | **5.9E-81** |
| **150** | **34702** | **P** | **150** | **67397** | **5.9E-81** |
| **150** | **43004** | **P** | **150** | **45140** | **5.9E-81** |
| **150** | **43004** | **P** | **150** | **204875** | **5.9E-81** |
| **150** | **49800** | **P** | **150** | **78078** | **5.9E-81** |
| **149** | **78227** | **P** | **149** | **179873** | **2.36E-80** |
| **149** | **146112** | **F** | **149** | **170514** | **2.36E-80** |
| **149** | **180017** | **F** | **149** | **199797** | **2.36E-80** |
| **148** | **9316** | **F** | **148** | **126547** | **9.44E-80** |
| **148** | **97308** | **F** | **148** | **97463** | **9.44E-80** |
| **147** | **108640** | **F** | **147** | **152562** | **3.78E-79** |
| **147** | **180911** | **F** | **147** | **181058** | **3.78E-79** |
| **146** | **117636** | **P** | **146** | **134341** | **1.51E-78** |
| **145** | **3314** | **F** | **145** | **3459** | **6.04E-78** |
| **144** | **10765** | **F** | **144** | **10909** | **2.42E-77** |
| **144** | **86775** | **P** | **144** | **126986** | **2.42E-77** |
| **144** | **89182** | **F** | **144** | **89325** | **2.42E-77** |
| **144** | **149223** | **P** | **144** | **179731** | **2.42E-77** |
| **152** | **137843** | **F** | **152** | **146111** | **3.81E-77** |
| **148** | **14339** | **F** | **148** | **169691** | **4.19E-77** |
| **143** | **182428** | **F** | **143** | **182590** | **9.67E-77** |
| **142** | **50360** | **P** | **142** | **50853** | **3.87E-76** |
| **142** | **190814** | **F** | **142** | **190958** | **3.87E-76** |
| **149** | **137844** | **F** | **149** | **170514** | **2.34E-75** |
| **140** | **69774** | **F** | **140** | **69914** | **6.19E-75** |
| **139** | **162166** | **F** | **139** | **162305** | **2.47E-74** |
| **138** | **68756** | **F** | **138** | **135110** | **9.9E-74** |
| **138** | **177712** | **F** | **138** | **177851** | **9.9E-74** |
| **136** | **167099** | **P** | **136** | **168252** | **1.58E-72** |
| **135** | **88207** | **F** | **135** | **88342** | **6.34E-72** |
| **134** | **77884** | **P** | **134** | **158316** | **2.53E-71** |
| **128** | **111173** | **F** | **128** | **167264** | **1.04E-67** |
| **139** | **81580** | **F** | **139** | **111080** | **2.93E-67** |
| **127** | **73317** | **F** | **127** | **73444** | **4.15E-67** |
| **126** | **14370** | **F** | **126** | **171466** | **1.66E-66** |
| **126** | **128893** | **F** | **126** | **129019** | **1.66E-66** |
| **130** | **173802** | **P** | **130** | **176292** | **2.53E-66** |
| **128** | **43888** | **F** | **128** | **157777** | **3.99E-65** |
| **122** | **111301** | **P** | **122** | **147822** | **4.25E-64** |
| **121** | **55524** | **F** | **121** | **193417** | **1.7E-63** |
| **118** | **34734** | **F** | **118** | **196863** | **1.09E-61** |
| **116** | **169661** | **F** | **116** | **171352** | **1.74E-60** |
| **115** | **43901** | **F** | **115** | **157790** | **6.97E-60** |
| **115** | **81720** | **F** | **115** | **176530** | **6.97E-60** |
| **117** | **169722** | **F** | **117** | **171466** | **1.53E-58** |
| **112** | **14032** | **F** | **112** | **206530** | **4.46E-58** |
| **112** | **169381** | **F** | **112** | **206530** | **4.46E-58** |
| **119** | **146106** | **P** | **119** | **194449** | **1.72E-57** |
| **121** | **103641** | **P** | **121** | **146140** | **1.32E-56** |
| **121** | **103641** | **P** | **121** | **170542** | **1.32E-56** |
| **108** | **25565** | **F** | **108** | **130423** | **1.14E-55** |
| **108** | **185646** | **F** | **108** | **185754** | **1.14E-55** |
| **112** | **160266** | **F** | **112** | **168607** | **1.5E-55** |
| **107** | **4766** | **P** | **107** | **146106** | **4.57E-55** |
| **114** | **137843** | **P** | **114** | **194449** | **1.62E-54** |
| **113** | **4766** | **F** | **113** | **194461** | **6.35E-54** |
| **113** | **170514** | **P** | **113** | **194449** | **6.35E-54** |
| **102** | **4664** | **P** | **102** | **137900** | **4.67E-52** |
| **102** | **4766** | **P** | **102** | **137843** | **4.67E-52** |
| **102** | **39630** | **F** | **102** | **40448** | **4.67E-52** |
| **101** | **4766** | **P** | **101** | **170514** | **1.87E-51** |
| **110** | **103652** | **P** | **110** | **137872** | **4.16E-50** |
| **98** | **160169** | **F** | **98** | **160266** | **1.2E-49** |
| **97** | **64734** | **P** | **97** | **180069** | **4.79E-49** |
| **101** | **160166** | **F** | **101** | **168604** | **5.67E-49** |
| **96** | **103690** | **F** | **96** | **107458** | **1.91E-48** |
| **95** | **33133** | **F** | **95** | **33230** | **7.66E-48** |
| **95** | **160283** | **F** | **95** | **168624** | **7.66E-48** |
| **94** | **178083** | **F** | **94** | **178176** | **3.06E-47** |
| **98** | **3361** | **P** | **98** | **16371** | **3.52E-47** |
| **97** | **25359** | **P** | **97** | **173279** | **1.39E-46** |
| **91** | **103746** | **F** | **91** | **103872** | **1.96E-45** |
| **90** | **744** | **P** | **90** | **18978** | **7.84E-45** |
| **90** | **11705** | **P** | **90** | **24772** | **7.84E-45** |
| **90** | **21404** | **P** | **90** | **137557** | **7.84E-45** |
| **90** | **43028** | **P** | **90** | **137557** | **7.84E-45** |
| **90** | **45176** | **F** | **90** | **137557** | **7.84E-45** |
| **90** | **58891** | **P** | **90** | **116920** | **7.84E-45** |
| **90** | **137557** | **F** | **90** | **204911** | **7.84E-45** |
| **89** | **24634** | **F** | **89** | **150767** | **3.14E-44** |
| **88** | **137748** | **F** | **88** | **146103** | **1.25E-43** |
| **95** | **4671** | **P** | **95** | **146168** | **3.08E-43** |
| **86** | **14339** | **F** | **86** | **171382** | **2.01E-42** |
| **93** | **4673** | **F** | **93** | **103641** | **4.72E-42** |
| **93** | **4673** | **P** | **93** | **170570** | **4.72E-42** |
| **85** | **4788** | **P** | **85** | **137751** | **8.03E-42** |
| **84** | **122678** | **F** | **84** | **122760** | **3.21E-41** |
| **91** | **89169** | **P** | **91** | **168741** | **7.23E-41** |
| **82** | **4684** | **P** | **82** | **146168** | **5.14E-40** |
| **85** | **103637** | **F** | **85** | **107371** | **2.05E-39** |
| **81** | **4798** | **P** | **81** | **15177** | **2.06E-39** |
| **81** | **160186** | **F** | **81** | **168624** | **2.06E-39** |
| **84** | **66292** | **P** | **84** | **198031** | **8.1E-39** |
| **84** | **182702** | **P** | **84** | **198031** | **8.1E-39** |
| **80** | **137756** | **F** | **80** | **137843** | **8.22E-39** |
| **79** | **137757** | **F** | **79** | **170514** | **3.29E-38** |
| **86** | **15172** | **P** | **86** | **194493** | **6.6E-38** |
| **85** | **137751** | **P** | **85** | **194483** | **2.58E-37** |
| **81** | **107375** | **P** | **81** | **146180** | **5E-37** |
| **81** | **107375** | **P** | **81** | **170582** | **5E-37** |
| **76** | **198151** | **F** | **76** | **201241** | **2.11E-36** |
| **75** | **15183** | **F** | **75** | **137751** | **8.42E-36** |
| **75** | **15183** | **F** | **75** | **146106** | **8.42E-36** |
| **85** | **103677** | **F** | **85** | **194449** | **2.14E-35** |
| **74** | **66249** | **F** | **74** | **182497** | **3.37E-35** |
| **73** | **13255** | **F** | **73** | **68405** | **1.35E-34** |
| **71** | **24452** | **F** | **71** | **154075** | **2.16E-33** |
| **78** | **89325** | **P** | **78** | **168741** | **3.56E-33** |
| **81** | **4673** | **F** | **81** | **107375** | **4.74E-33** |
| **81** | **107375** | **P** | **81** | **137912** | **4.74E-33** |
| **70** | **15188** | **F** | **70** | **137843** | **8.62E-33** |
| **69** | **15189** | **F** | **69** | **170514** | **3.45E-32** |
| **69** | **127644** | **F** | **69** | **127711** | **3.45E-32** |
| **69** | **182461** | **P** | **69** | **205669** | **3.45E-32** |
| **69** | **182623** | **P** | **69** | **205669** | **3.45E-32** |
| **68** | **46649** | **F** | **68** | **205671** | **1.38E-31** |
| **75** | **7957** | **P** | **75** | **108677** | **2.1E-31** |
| **75** | **7957** | **P** | **75** | **152599** | **2.1E-31** |
| **71** | **69749** | **F** | **71** | **103912** | **4.59E-31** |
| **71** | **89189** | **P** | **71** | **168741** | **4.59E-31** |
| **67** | **12138** | **F** | **67** | **12208** | **5.52E-31** |
| **67** | **46649** | **P** | **67** | **182461** | **5.52E-31** |
| **67** | **46649** | **P** | **67** | **182623** | **5.52E-31** |
| **67** | **143994** | **F** | **67** | **163607** | **5.52E-31** |
| **68** | **81578** | **F** | **68** | **180580** | **2.81E-29** |
| **68** | **137889** | **P** | **68** | **194449** | **2.81E-29** |
| **68** | **146157** | **P** | **68** | **194449** | **2.81E-29** |
| **68** | **170559** | **P** | **68** | **194449** | **2.81E-29** |
| **63** | **24523** | **F** | **63** | **154145** | **1.41E-28** |
| **70** | **40344** | **F** | **70** | **147929** | **1.87E-28** |
| **73** | **4766** | **F** | **73** | **103689** | **2.26E-28** |
| **62** | **45893** | **P** | **62** | **182468** | **5.65E-28** |
| **62** | **45893** | **P** | **62** | **182630** | **5.65E-28** |
| **62** | **81720** | **P** | **62** | **173557** | **5.65E-28** |
| **72** | **4767** | **F** | **72** | **107458** | **8.68E-28** |
| **72** | **107458** | **P** | **72** | **137872** | **8.68E-28** |
| **72** | **107458** | **P** | **72** | **146140** | **8.68E-28** |
| **72** | **107458** | **P** | **72** | **170542** | **8.68E-28** |
| **72** | **107458** | **F** | **72** | **194462** | **8.68E-28** |
| **65** | **4814** | **F** | **65** | **194509** | **1.72E-27** |
| **71** | **111075** | **F** | **71** | **180577** | **3.33E-27** |
| **64** | **111576** | **P** | **64** | **143662** | **6.78E-27** |
| **64** | **197977** | **F** | **64** | **198117** | **6.78E-27** |
| **60** | **45895** | **F** | **60** | **46649** | **9.04E-27** |
| **59** | **13601** | **P** | **59** | **33159** | **3.62E-26** |
| **59** | **13601** | **P** | **59** | **33256** | **3.62E-26** |
| **59** | **33159** | **P** | **59** | **168950** | **3.62E-26** |
| **59** | **33256** | **P** | **59** | **168950** | **3.62E-26** |
| **59** | **137898** | **P** | **59** | **194449** | **3.62E-26** |
| **59** | **146166** | **P** | **59** | **194449** | **3.62E-26** |
| **59** | **170568** | **P** | **59** | **194449** | **3.62E-26** |
| **57** | **4709** | **F** | **57** | **194449** | **5.79E-25** |
| **57** | **7957** | **F** | **57** | **206665** | **5.79E-25** |
| **57** | **108695** | **P** | **57** | **206665** | **5.79E-25** |
| **57** | **123343** | **F** | **57** | **123400** | **5.79E-25** |
| **57** | **152617** | **P** | **57** | **206665** | **5.79E-25** |
| **56** | **4823** | **F** | **56** | **194518** | **2.31E-24** |
| **56** | **59117** | **P** | **56** | **153300** | **2.31E-24** |
| **56** | **104358** | **F** | **56** | **104412** | **2.31E-24** |
| **56** | **137693** | **P** | **56** | **160313** | **2.31E-24** |
| **56** | **137693** | **P** | **56** | **168654** | **2.31E-24** |
| **55** | **171413** | **F** | **55** | **171466** | **9.26E-24** |
| **62** | **111647** | **P** | **62** | **143593** | **9.62E-24** |
| **54** | **33057** | **P** | **54** | **104008** | **3.7E-23** |
| **53** | **15258** | **F** | **53** | **137949** | **1.48E-22** |
| **53** | **69831** | **F** | **53** | **182489** | **1.48E-22** |
| **53** | **69831** | **F** | **53** | **182651** | **1.48E-22** |
| **53** | **69971** | **F** | **53** | **182489** | **1.48E-22** |
| **53** | **69971** | **F** | **53** | **182651** | **1.48E-22** |
| **63** | **71847** | **P** | **63** | **145896** | **1.51E-22** |
| **52** | **4786** | **P** | **52** | **89256** | **5.93E-22** |
| **52** | **4786** | **P** | **52** | **89399** | **5.93E-22** |
| **52** | **89256** | **F** | **52** | **137873** | **5.93E-22** |
| **52** | **89256** | **F** | **52** | **146141** | **5.93E-22** |
| **52** | **89256** | **F** | **52** | **170543** | **5.93E-22** |
| **52** | **89399** | **F** | **52** | **137873** | **5.93E-22** |
| **52** | **89399** | **F** | **52** | **146141** | **5.93E-22** |
| **52** | **89399** | **F** | **52** | **170543** | **5.93E-22** |
| **52** | **160207** | **F** | **52** | **182453** | **5.93E-22** |
| **52** | **160207** | **F** | **52** | **182615** | **5.93E-22** |
| **52** | **160304** | **F** | **52** | **182453** | **5.93E-22** |
| **52** | **160304** | **F** | **52** | **182615** | **5.93E-22** |
| **52** | **168645** | **F** | **52** | **182453** | **5.93E-22** |
| **52** | **168645** | **F** | **52** | **182615** | **5.93E-22** |
| **52** | **169609** | **F** | **52** | **182574** | **5.93E-22** |
| **55** | **111091** | **F** | **55** | **180593** | **1.53E-21** |
| **51** | **137698** | **P** | **51** | **160216** | **2.37E-21** |
| **54** | **66268** | **P** | **54** | **137651** | **6E-21** |
| **54** | **137651** | **P** | **54** | **182516** | **6E-21** |
| **54** | **137651** | **P** | **54** | **182678** | **6E-21** |
| **50** | **14853** | **F** | **50** | **160019** | **9.48E-21** |
| **50** | **89256** | **F** | **50** | **137786** | **9.48E-21** |
| **50** | **89399** | **F** | **50** | **137786** | **9.48E-21** |
| **50** | **160019** | **F** | **50** | **170208** | **9.48E-21** |
| **49** | **25407** | **P** | **49** | **173279** | **3.79E-20** |
| **49** | **75887** | **F** | **49** | **75936** | **3.79E-20** |
| **52** | **13407** | **P** | **52** | **20589** | **9.24E-20** |
| **51** | **69749** | **F** | **51** | **103786** | **3.63E-19** |
| **47** | **14418** | **F** | **47** | **66329** | **6.07E-19** |
| **47** | **14418** | **F** | **47** | **182739** | **6.07E-19** |
| **47** | **40367** | **P** | **47** | **111242** | **6.07E-19** |
| **47** | **40367** | **P** | **47** | **167333** | **6.07E-19** |
| **47** | **66329** | **F** | **47** | **169770** | **6.07E-19** |
| **47** | **66329** | **F** | **47** | **171514** | **6.07E-19** |
| **47** | **169770** | **F** | **47** | **182739** | **6.07E-19** |
| **47** | **171514** | **F** | **47** | **182739** | **6.07E-19** |
| **50** | **21480** | **P** | **50** | **69644** | **1.42E-18** |
| **50** | **43104** | **P** | **50** | **69644** | **1.42E-18** |
| **50** | **45140** | **F** | **50** | **69644** | **1.42E-18** |
| **50** | **69644** | **F** | **50** | **204875** | **1.42E-18** |
| **56** | **89256** | **P** | **56** | **194477** | **1.73E-18** |
| **56** | **89399** | **P** | **56** | **194477** | **1.73E-18** |
| **46** | **29644** | **F** | **46** | **29693** | **2.43E-18** |
| **46** | **69914** | **F** | **46** | **103937** | **2.43E-18** |
| **46** | **81673** | **F** | **46** | **167264** | **2.43E-18** |
| **49** | **14418** | **P** | **49** | **198029** | **5.57E-18** |
| **49** | **169770** | **P** | **49** | **198029** | **5.57E-18** |
| **49** | **171514** | **P** | **49** | **198029** | **5.57E-18** |
| **45** | **4721** | **F** | **45** | **4766** | **9.71E-18** |
| **45** | **66249** | **F** | **45** | **69839** | **9.71E-18** |
| **45** | **66249** | **F** | **45** | **69979** | **9.71E-18** |
| **48** | **22035** | **F** | **48** | **35625** | **2.18E-17** |
| **48** | **35625** | **P** | **48** | **44522** | **2.18E-17** |
| **48** | **35625** | **F** | **48** | **60538** | **2.18E-17** |
| **48** | **35625** | **P** | **48** | **204258** | **2.18E-17** |
| **44** | **4722** | **F** | **44** | **107458** | **3.88E-17** |
| **44** | **13327** | **P** | **44** | **89159** | **3.88E-17** |
| **44** | **19843** | **P** | **44** | **45895** | **3.88E-17** |
| **44** | **19843** | **F** | **44** | **182484** | **3.88E-17** |
| **44** | **19843** | **F** | **44** | **182646** | **3.88E-17** |
| **44** | **19843** | **P** | **44** | **205671** | **3.88E-17** |
| **44** | **46672** | **P** | **44** | **160215** | **3.88E-17** |
| **44** | **46672** | **P** | **44** | **160312** | **3.88E-17** |
| **44** | **46672** | **P** | **44** | **168653** | **3.88E-17** |
| **44** | **46674** | **F** | **44** | **103979** | **3.88E-17** |
| **44** | **160215** | **P** | **44** | **205694** | **3.88E-17** |
| **44** | **160312** | **P** | **44** | **205694** | **3.88E-17** |
| **44** | **168653** | **P** | **44** | **205694** | **3.88E-17** |
| **43** | **46672** | **F** | **43** | **137706** | **1.55E-16** |
| **43** | **103979** | **F** | **43** | **205696** | **1.55E-16** |
| **43** | **137706** | **P** | **43** | **182462** | **1.55E-16** |
| **43** | **137706** | **P** | **43** | **182624** | **1.55E-16** |
| **43** | **137706** | **F** | **43** | **205694** | **1.55E-16** |
| **52** | **89256** | **P** | **52** | **103709** | **3.54E-16** |
| **52** | **89256** | **P** | **52** | **107477** | **3.54E-16** |
| **52** | **89399** | **P** | **52** | **103709** | **3.54E-16** |
| **52** | **89399** | **P** | **52** | **107477** | **3.54E-16** |
| **42** | **45148** | **F** | **42** | **69652** | **6.21E-16** |
| **42** | **69652** | **F** | **42** | **204883** | **6.21E-16** |
| **42** | **103979** | **P** | **42** | **160215** | **6.21E-16** |
| **42** | **103979** | **P** | **42** | **160312** | **6.21E-16** |
| **42** | **103979** | **P** | **42** | **168653** | **6.21E-16** |
| **42** | **103979** | **P** | **42** | **182461** | **6.21E-16** |
| **42** | **103979** | **P** | **42** | **182623** | **6.21E-16** |
| **45** | **103872** | **F** | **45** | **107514** | **1.31E-15** |
| **45** | **107411** | **F** | **45** | **194449** | **1.31E-15** |
| **51** | **103711** | **P** | **51** | **137785** | **1.33E-15** |
| **51** | **107479** | **P** | **51** | **137785** | **1.33E-15** |
| **41** | **19846** | **F** | **41** | **180917** | **2.49E-15** |
| **41** | **19846** | **F** | **41** | **181064** | **2.49E-15** |
| **41** | **42373** | **P** | **41** | **176714** | **2.49E-15** |
| **41** | **45893** | **P** | **41** | **69831** | **2.49E-15** |
| **41** | **45893** | **P** | **41** | **69971** | **2.49E-15** |
| **41** | **45895** | **P** | **41** | **180917** | **2.49E-15** |
| **41** | **45895** | **P** | **41** | **181064** | **2.49E-15** |
| **41** | **46649** | **P** | **41** | **180917** | **2.49E-15** |
| **41** | **46649** | **P** | **41** | **181064** | **2.49E-15** |
| **41** | **69831** | **P** | **41** | **205669** | **2.49E-15** |
| **41** | **69971** | **P** | **41** | **205669** | **2.49E-15** |
| **41** | **103979** | **F** | **41** | **137708** | **2.49E-15** |
| **41** | **112805** | **P** | **41** | **144859** | **2.49E-15** |
| **41** | **180917** | **F** | **41** | **182487** | **2.49E-15** |
| **41** | **180917** | **F** | **41** | **182649** | **2.49E-15** |
| **41** | **180917** | **P** | **41** | **205671** | **2.49E-15** |
| **41** | **181064** | **F** | **41** | **182487** | **2.49E-15** |
| **41** | **181064** | **F** | **41** | **182649** | **2.49E-15** |
| **41** | **181064** | **P** | **41** | **205671** | **2.49E-15** |
| **47** | **111242** | **P** | **47** | **147952** | **5.9E-15** |
| **47** | **147952** | **P** | **47** | **167333** | **5.9E-15** |
| **40** | **4392** | **F** | **40** | **4431** | **9.94E-15** |
| **40** | **4392** | **P** | **40** | **15504** | **9.94E-15** |
| **40** | **4431** | **P** | **40** | **15543** | **9.94E-15** |
| **40** | **7974** | **P** | **40** | **108655** | **9.94E-15** |
| **40** | **7974** | **P** | **40** | **152577** | **9.94E-15** |
| **40** | **15151** | **P** | **40** | **160382** | **9.94E-15** |
| **40** | **15218** | **F** | **40** | **89256** | **9.94E-15** |
| **40** | **15218** | **F** | **40** | **89399** | **9.94E-15** |
| **40** | **15504** | **F** | **40** | **15543** | **9.94E-15** |
| **40** | **25220** | **F** | **40** | **174459** | **9.94E-15** |
| **40** | **108655** | **F** | **40** | **108695** | **9.94E-15** |
| **40** | **108655** | **F** | **40** | **152617** | **9.94E-15** |
| **40** | **108655** | **P** | **40** | **206682** | **9.94E-15** |
| **40** | **108695** | **F** | **40** | **152577** | **9.94E-15** |
| **40** | **134283** | **P** | **40** | **176210** | **9.94E-15** |
| **40** | **152577** | **F** | **40** | **152617** | **9.94E-15** |
| **40** | **152577** | **P** | **40** | **206682** | **9.94E-15** |
| **49** | **173369** | **P** | **49** | **173369** | **1.89E-14** |
| **43** | **40371** | **F** | **43** | **147956** | **2E-14** |
| **46** | **15258** | **F** | **46** | **146217** | **2.26E-14** |
| **39** | **19848** | **F** | **39** | **69831** | **3.98E-14** |
| **39** | **19848** | **F** | **39** | **69971** | **3.98E-14** |
| **39** | **46649** | **P** | **39** | **69831** | **3.98E-14** |
| **39** | **46649** | **P** | **39** | **69971** | **3.98E-14** |
| **39** | **69831** | **F** | **39** | **180919** | **3.98E-14** |
| **39** | **69831** | **F** | **39** | **181066** | **3.98E-14** |
| **39** | **69971** | **F** | **39** | **180919** | **3.98E-14** |
| **39** | **69971** | **F** | **39** | **181066** | **3.98E-14** |
| **48** | **36630** | **F** | **48** | **122098** | **7.08E-14** |
| **38** | **172741** | **F** | **38** | **198145** | **1.59E-13** |
| **44** | **15258** | **P** | **44** | **103641** | **3.31E-13** |
| **44** | **15258** | **P** | **44** | **107375** | **3.31E-13** |
| **44** | **15258** | **F** | **44** | **170619** | **3.31E-13** |
| **37** | **13422** | **P** | **37** | **20589** | **6.36E-13** |
| **37** | **45918** | **F** | **37** | **137706** | **6.36E-13** |
| **37** | **45918** | **P** | **37** | **160222** | **6.36E-13** |
| **37** | **45918** | **P** | **37** | **160319** | **6.36E-13** |
| **37** | **45918** | **P** | **37** | **168660** | **6.36E-13** |
| **37** | **101810** | **F** | **37** | **101843** | **6.36E-13** |
| **36** | **169625** | **F** | **36** | **182428** | **2.55E-12** |
| **35** | **14456** | **F** | **35** | **47992** | **1.02E-11** |
| **35** | **15146** | **P** | **35** | **107557** | **1.02E-11** |
| **35** | **45920** | **F** | **35** | **103979** | **1.02E-11** |
| **35** | **47992** | **F** | **35** | **171552** | **1.02E-11** |
| **35** | **111250** | **P** | **35** | **147956** | **1.02E-11** |
| **35** | **137670** | **P** | **35** | **182516** | **1.02E-11** |
| **35** | **137670** | **P** | **35** | **182678** | **1.02E-11** |
| **38** | **167377** | **F** | **38** | **176214** | **1.81E-11** |
| **34** | **89078** | **F** | **34** | **145896** | **4.07E-11** |
| **43** | **95361** | **P** | **43** | **136555** | **5.18E-11** |
| **40** | **89272** | **P** | **40** | **194477** | **6.98E-11** |
| **40** | **89415** | **P** | **40** | **194477** | **6.98E-11** |
| **33** | **834** | **P** | **33** | **18908** | **1.63E-10** |
| **33** | **8031** | **F** | **33** | **46682** | **1.63E-10** |
| **33** | **8031** | **F** | **33** | **103987** | **1.63E-10** |
| **33** | **8031** | **F** | **33** | **137716** | **1.63E-10** |
| **33** | **8031** | **P** | **33** | **160216** | **1.63E-10** |
| **33** | **8031** | **P** | **33** | **160313** | **1.63E-10** |
| **33** | **8031** | **P** | **33** | **168654** | **1.63E-10** |
| **33** | **8031** | **P** | **33** | **182462** | **1.63E-10** |
| **33** | **8031** | **P** | **33** | **182624** | **1.63E-10** |
| **33** | **8031** | **F** | **33** | **205704** | **1.63E-10** |
| **33** | **45893** | **P** | **33** | **66249** | **1.63E-10** |
| **33** | **58170** | **F** | **33** | **58202** | **1.63E-10** |
| **33** | **66249** | **P** | **33** | **205669** | **1.63E-10** |
| **33** | **98300** | **F** | **33** | **98332** | **1.63E-10** |
| **33** | **147975** | **P** | **33** | **176660** | **1.63E-10** |
| **36** | **134283** | **P** | **36** | **167377** | **2.75E-10** |
| **36** | **172741** | **F** | **36** | **198005** | **2.75E-10** |
| **32** | **8175** | **P** | **32** | **66297** | **6.52E-10** |
| **32** | **8175** | **P** | **32** | **182707** | **6.52E-10** |
| **32** | **8175** | **F** | **32** | **198078** | **6.52E-10** |
| **32** | **13433** | **P** | **32** | **145852** | **6.52E-10** |
| **32** | **137693** | **F** | **32** | **180308** | **6.52E-10** |
| **32** | **160337** | **P** | **32** | **180308** | **6.52E-10** |
| **32** | **168678** | **P** | **32** | **180308** | **6.52E-10** |
| **32** | **172747** | **F** | **32** | **201241** | **6.52E-10** |
| **41** | **15217** | **P** | **41** | **103721** | **7.15E-10** |
| **41** | **15217** | **P** | **41** | **107489** | **7.15E-10** |
| **35** | **68891** | **F** | **35** | **146811** | **1.07E-09** |
| **31** | **19856** | **F** | **31** | **66249** | **2.61E-09** |
| **31** | **46649** | **P** | **31** | **66249** | **2.61E-09** |
| **31** | **66249** | **F** | **31** | **180927** | **2.61E-09** |
| **31** | **66249** | **F** | **31** | **181074** | **2.61E-09** |
| **31** | **182540** | **P** | **31** | **198084** | **2.61E-09** |
| **40** | **89272** | **P** | **40** | **103705** | **2.65E-09** |
| **40** | **89272** | **P** | **40** | **107473** | **2.65E-09** |
| **40** | **89415** | **P** | **40** | **103705** | **2.65E-09** |
| **40** | **89415** | **P** | **40** | **107473** | **2.65E-09** |
| **37** | **57914** | **F** | **37** | **139048** | **3.81E-09** |
| **34** | **8286** | **F** | **34** | **21478** | **4.15E-09** |
| **34** | **8286** | **F** | **34** | **43102** | **4.15E-09** |
| **34** | **8286** | **P** | **34** | **45158** | **4.15E-09** |
| **34** | **8286** | **P** | **34** | **204893** | **4.15E-09** |
| **34** | **13327** | **F** | **34** | **168798** | **4.15E-09** |
| **34** | **137802** | **P** | **34** | **194483** | **4.15E-09** |
| **39** | **4608** | **F** | **39** | **96625** | **9.81E-09** |
| **39** | **15328** | **P** | **39** | **96625** | **9.81E-09** |
| **39** | **135022** | **P** | **39** | **135022** | **9.81E-09** |
| **33** | **4766** | **F** | **33** | **107423** | **1.61E-08** |
| **35** | **95369** | **P** | **35** | **136555** | **5.45E-08** |
| **32** | **8288** | **P** | **32** | **69662** | **6.25E-08** |
| **32** | **21497** | **P** | **32** | **168578** | **6.25E-08** |
| **32** | **21497** | **F** | **32** | **178568** | **6.25E-08** |
| **32** | **43121** | **P** | **32** | **168578** | **6.25E-08** |
| **32** | **43121** | **F** | **32** | **178568** | **6.25E-08** |
| **32** | **45141** | **F** | **32** | **168578** | **6.25E-08** |
| **32** | **45141** | **P** | **32** | **178568** | **6.25E-08** |
| **32** | **107424** | **F** | **32** | **107458** | **6.25E-08** |
| **32** | **111715** | **P** | **32** | **143546** | **6.25E-08** |
| **32** | **168578** | **F** | **32** | **204876** | **6.25E-08** |
| **32** | **178568** | **P** | **32** | **204876** | **6.25E-08** |
| **34** | **64446** | **P** | **34** | **186870** | **0.000000206** |
| **34** | **186870** | **F** | **34** | **200200** | **0.000000206** |
| **31** | **1530** | **F** | **31** | **89557** | **0.000000242** |
| **31** | **18277** | **P** | **31** | **89557** | **0.000000242** |
| **31** | **28322** | **P** | **31** | **108370** | **0.000000242** |
| **31** | **45924** | **F** | **31** | **146067** | **0.000000242** |
| **31** | **46678** | **F** | **31** | **146067** | **0.000000242** |
| **31** | **47992** | **F** | **31** | **169808** | **0.000000242** |
| **31** | **71978** | **F** | **31** | **72011** | **0.000000242** |
| **31** | **89281** | **P** | **31** | **194477** | **0.000000242** |
| **31** | **89424** | **P** | **31** | **194477** | **0.000000242** |
| **31** | **103983** | **F** | **31** | **146067** | **0.000000242** |
| **31** | **111260** | **P** | **31** | **134310** | **0.000000242** |
| **31** | **134310** | **P** | **31** | **167351** | **0.000000242** |
| **31** | **137712** | **F** | **31** | **146067** | **0.000000242** |
| **31** | **146067** | **P** | **31** | **160222** | **0.000000242** |
| **31** | **146067** | **P** | **31** | **160319** | **0.000000242** |
| **31** | **146067** | **P** | **31** | **168660** | **0.000000242** |
| **31** | **146067** | **P** | **31** | **182468** | **0.000000242** |
| **31** | **146067** | **P** | **31** | **182630** | **0.000000242** |
| **31** | **146067** | **F** | **31** | **205700** | **0.000000242** |
| **31** | **168581** | **P** | **31** | **178566** | **0.000000242** |
| **36** | **42352** | **P** | **36** | **167400** | **0.000000491** |
| **36** | **46871** | **F** | **36** | **46889** | **0.000000491** |
| **35** | **71876** | **P** | **35** | **89077** | **0.0000018** |
| **32** | **69645** | **F** | **32** | **168578** | **0.00000291** |
| **32** | **69645** | **P** | **32** | **178568** | **0.00000291** |
| **32** | **89280** | **P** | **32** | **103705** | **0.00000291** |
| **32** | **89280** | **P** | **32** | **107473** | **0.00000291** |
| **32** | **89423** | **P** | **32** | **103705** | **0.00000291** |
| **32** | **89423** | **P** | **32** | **107473** | **0.00000291** |
| **31** | **3454** | **P** | **31** | **16462** | **0.0000109** |
| **31** | **8166** | **P** | **31** | **13342** | **0.0000109** |
| **31** | **19846** | **P** | **31** | **33111** | **0.0000109** |
| **31** | **21498** | **P** | **31** | **49802** | **0.0000109** |
| **31** | **21498** | **F** | **31** | **78195** | **0.0000109** |
| **31** | **22556** | **F** | **31** | **61056** | **0.0000109** |
| **31** | **33111** | **F** | **31** | **45905** | **0.0000109** |
| **31** | **33111** | **F** | **31** | **46659** | **0.0000109** |
| **31** | **33111** | **P** | **31** | **180917** | **0.0000109** |
| **31** | **33111** | **P** | **31** | **181064** | **0.0000109** |
| **31** | **33111** | **P** | **31** | **182487** | **0.0000109** |
| **31** | **33111** | **P** | **31** | **182649** | **0.0000109** |
| **31** | **33111** | **F** | **31** | **205681** | **0.0000109** |
| **31** | **43124** | **P** | **31** | **49800** | **0.0000109** |
| **31** | **45141** | **F** | **31** | **49802** | **0.0000109** |
| **31** | **45141** | **P** | **31** | **78195** | **0.0000109** |
| **31** | **49802** | **F** | **31** | **204876** | **0.0000109** |
| **31** | **78195** | **P** | **31** | **204876** | **0.0000109** |
| **31** | **78199** | **P** | **31** | **168574** | **0.0000109** |
| **33** | **69670** | **F** | **33** | **103754** | **0.000024** |
| **33** | **69670** | **F** | **33** | **103880** | **0.000024** |
| **32** | **40382** | **P** | **32** | **176669** | **0.0000873** |
| **32** | **69670** | **F** | **32** | **107522** | **0.0000873** |
| **32** | **76119** | **F** | **32** | **76123** | **0.0000873** |
| **32** | **102969** | **F** | **32** | **103014** | **0.0000873** |
| **32** | **111242** | **F** | **32** | **176669** | **0.0000873** |
| **32** | **167333** | **F** | **32** | **176669** | **0.0000873** |
| **31** | **7973** | **F** | **31** | **33118** | **0.000316** |
| **31** | **8181** | **P** | **31** | **182540** | **0.000316** |
| **31** | **33118** | **P** | **31** | **108705** | **0.000316** |
| **31** | **33118** | **P** | **31** | **152627** | **0.000316** |
| **31** | **33118** | **F** | **31** | **206681** | **0.000316** |
| **31** | **49802** | **F** | **31** | **69645** | **0.000316** |
| **31** | **49802** | **F** | **31** | **168578** | **0.000316** |
| **31** | **49802** | **P** | **31** | **178569** | **0.000316** |
| **31** | **51489** | **F** | **31** | **131609** | **0.000316** |
| **31** | **65104** | **P** | **31** | **140246** | **0.000316** |
| **31** | **69645** | **P** | **31** | **78195** | **0.000316** |
| **31** | **69853** | **P** | **31** | **137679** | **0.000316** |
| **31** | **69993** | **P** | **31** | **137679** | **0.000316** |
| **31** | **78199** | **F** | **31** | **178573** | **0.000316** |
| **31** | **78200** | **P** | **31** | **160136** | **0.000316** |
| **31** | **89118** | **F** | **31** | **145949** | **0.000316** |
| **31** | **140246** | **F** | **31** | **199342** | **0.000316** |
| **31** | **160240** | **P** | **31** | **180309** | **0.000316** |
| **Forward (direct) show [F], Reverse show [R]** | | | | | |
