# Supplementary material for: Assembly and comparative analysis of the complete mitochondrial genome of Isopyrum anemonoides (Ranunculaceae)
Source: PLoS One. 2023 Oct 5;18(10):e0286628. doi: 10.1371/journal.pone.0286628 (PMC10553351; doi:10.1371/journal.pone.0286628)
Supplement: S4 Table — (DOCX) [file pone.0286628.s004.docx]

**S4 Table. Tandem repeats detected in the mitogenomes of *Isopyrum anemonoides***

| **NO.** | **Indices** | **Period Size** | **Copy Number** | **Consensus Size** | **Percent Matches** | **Percent Indels** | **Score** | **Entropy (0-2)** |
| --- | --- | --- | --- | --- | --- | --- | --- | --- |
| 1 | 3315--3604 | 145 | 2 | 145 | 100 | 0 | 580 | 1.94 |
| 2 | 4393--4471 | 39 | 2 | 39 | 100 | 0 | 158 | 1.97 |
| 3 | 4722--4811 | 45 | 2 | 45 | 100 | 0 | 180 | 1.95 |
| 4 | 10766--11053 | 144 | 2 | 144 | 100 | 0 | 576 | 1.89 |
| 5 | 12139--12275 | 70 | 2 | 70 | 100 | 0 | 274 | 1.99 |
| 6 | 15505--15583 | 39 | 2 | 39 | 100 | 0 | 158 | 1.97 |
| 7 | 19042--19095 | 27 | 2 | 27 | 100 | 0 | 108 | 1.91 |
| 8 | 29645--29739 | 49 | 1.9 | 49 | 100 | 0 | 190 | 1.98 |
| 9 | 33134--33325 | 97 | 2 | 97 | 100 | 0 | 384 | 1.99 |
| 10 | 35053--35090 | 19 | 2 | 19 | 100 | 0 | 76 | 1.85 |
| 11 | 46872--46925 | 18 | 3 | 18 | 91 | 0 | 90 | 1.72 |
| 12 | 58171--58235 | 32 | 2 | 32 | 100 | 0 | 130 | 1.71 |
| 13 | 69775--70054 | 140 | 2 | 140 | 100 | 0 | 560 | 1.99 |
| 14 | 73318--73571 | 127 | 2 | 127 | 100 | 0 | 508 | 1.97 |
| 15 | 75888--75985 | 49 | 2 | 49 | 100 | 0 | 196 | 1.94 |
| 16 | 88208--88477 | 135 | 2 | 135 | 100 | 0 | 540 | 1.97 |
| 17 | 89183--89469 | 143 | 2 | 143 | 100 | 0 | 574 | 1.99 |
| 18 | 97309--97611 | 155 | 2 | 155 | 100 | 0 | 606 | 2 |
| 19 | 98294--98365 | 32 | 2.3 | 32 | 95 | 4 | 130 | 1.97 |
| 20 | 101811--101880 | 33 | 2.1 | 33 | 100 | 0 | 140 | 1.82 |
| 21 | 104359--104468 | 54 | 2 | 54 | 100 | 0 | 220 | 1.78 |
| 23 | 107425--107490 | 34 | 1.9 | 34 | 96 | 0 | 123 | 1.92 |
| 24 | 108667--108730 | 20 | 3.2 | 20 | 65 | 30 | 55 | 1.9 |
| 25 | 108656--108735 | 40 | 2 | 40 | 100 | 0 | 160 | 1.89 |
| 26 | 122679--122844 | 82 | 2 | 82 | 100 | 0 | 332 | 1.92 |
| 27 | 123344--123457 | 57 | 2 | 57 | 100 | 0 | 228 | 1.91 |
| 28 | 124781--124830 | 24 | 2.1 | 24 | 100 | 0 | 100 | 1.91 |
| 29 | 127645--127780 | 67 | 2 | 67 | 100 | 0 | 272 | 1.96 |
| 30 | 128894--129145 | 126 | 2 | 126 | 100 | 0 | 504 | 1.99 |
| 31 | 135010--135047 | 15 | 2.5 | 15 | 86 | 0 | 58 | 1.86 |
| 32 | 137757--137923 | 87 | 1.9 | 87 | 100 | 0 | 334 | 1.98 |
| 33 | 140787--140822 | 18 | 2 | 18 | 100 | 0 | 72 | 1.72 |
| 34 | 141970--142001 | 16 | 2 | 16 | 100 | 0 | 64 | 2 |
| 35 | 148907--148966 | 30 | 2 | 30 | 100 | 0 | 120 | 1.76 |
| 36 | 152589--152652 | 20 | 3.2 | 20 | 65 | 30 | 55 | 1.9 |
| 37 | 152578--152657 | 40 | 2 | 40 | 100 | 0 | 160 | 1.89 |
| 38 | 154342--154373 | 16 | 2 | 16 | 93 | 0 | 55 | 1.32 |
| 39 | 154663--154689 | 13 | 2.1 | 13 | 100 | 0 | 54 | 1.54 |
| 40 | 160170--160364 | 97 | 2 | 97 | 100 | 0 | 390 | 1.98 |
| 41 | 162167--162444 | 139 | 2 | 139 | 100 | 0 | 556 | 1.97 |
| 42 | 171414--171521 | 53 | 2 | 53 | 100 | 0 | 216 | 1.86 |
| 43 | 177777--177825 | 24 | 2 | 24 | 92 | 0 | 80 | 1.69 |
| 44 | 177916--177964 | 24 | 2 | 24 | 92 | 0 | 80 | 1.69 |
| 45 | 177713--177989 | 139 | 2 | 139 | 100 | 0 | 554 | 1.91 |
| 46 | 178084--178270 | 93 | 2 | 93 | 100 | 0 | 374 | 1.98 |
| 47 | 180912--181205 | 147 | 2 | 147 | 100 | 0 | 588 | 2 |
| 48 | 182429--182733 | 162 | 1.9 | 162 | 100 | 0 | 610 | 2 |
| 49 | 183250--183292 | 18 | 2.4 | 18 | 81 | 18 | 54 | 1.96 |
| 50 | 185647--185862 | 108 | 2 | 108 | 100 | 0 | 432 | 1.9 |
| 51 | 189628--189677 | 24 | 2.1 | 24 | 100 | 0 | 100 | 1.95 |
| 52 | 190815--191100 | 144 | 2 | 144 | 100 | 0 | 572 | 1.96 |
