# Supplementary material for: Assembly and comparative analysis of the complete mitochondrial genome of Isopyrum anemonoides (Ranunculaceae)
Source: PLoS One. 2023 Oct 5;18(10):e0286628. doi: 10.1371/journal.pone.0286628 (PMC10553351; doi:10.1371/journal.pone.0286628)
Supplement: S5 Table — (DOCX) [file pone.0286628.s005.docx]

**S5 Table. SSRs in the mitochondrial genome of *Isopyrum anemonoides***

| **SSR nr.** | **size** | **start** | **end** | **SSR type** | **SSR** |
| --- | --- | --- | --- | --- | --- |
| 1 | 12 | 170 | 181 | p4 | (TATC)3 |
| 2 | 12 | 423 | 434 | p4 | (TTGC)3 |
| 3 | 12 | 496 | 507 | p4 | (TTGG)3 |
| 4 | 12 | 1915 | 1926 | p4 | (CATA)3 |
| 5 | 10 | 6190 | 6199 | p2 | (TA)5 |
| 6 | 11 | 7272 | 7282 | p1 | (A)11 |
| 7 | 10 | 8773 | 8782 | p1 | (T)10 |
| 8 | 16 | 11880 | 11895 | p1 | (A)16 |
| 9 | 12 | 13598 | 13609 | p4 | (ATGG)3 |
| 10 | 12 | 17877 | 17888 | p4 | (TATG)3 |
| 11 | 12 | 19333 | 19344 | p4 | (CCAA)3 |
| 12 | 12 | 19406 | 19417 | p4 | (GCAA)3 |
| 13 | 12 | 19659 | 19670 | p4 | (GATA)3 |
| 14 | 12 | 20700 | 20711 | p4 | (TAGC)3 |
| 15 | 12 | 21543 | 21554 | p4 | (TCAT)3 |
| 16 | 12 | 24651 | 24662 | p4 | (AAAG)3 |
| 17 | 11 | 24900 | 24910 | p1 | (A)11 |
| 18 | 15 | 25092 | 25106 | p5 | (GCCCG)3 |
| 19 | 11 | 25759 | 25769 | p1 | (T)11 |
| 20 | 12 | 31593 | 31604 | p1 | (A)12 |
| 21 | 12 | 35359 | 35370 | p2 | (TA)6 |
| 22 | 11 | 43570 | 43580 | p1 | (T)11 |
| 23 | 12 | 45117 | 45128 | p4 | (ATGA)3 |
| 24 | 11 | 49103 | 49113 | p1 | (T)11 |
| 25 | 10 | 49417 | 49426 | p2 | (AG)5 |
| 26 | 10 | 55453 | 55462 | p1 | (T)10 |
| 27 | 15 | 63784 | 63798 | p5 | (ATCGT)3 |
| 28 | 12 | 67325 | 67336 | p4 | (ACTG)3 |
| 29 | 12 | 71842 | 71853 | p3 | (CTA)4 |
| 30 | 11 | 72269 | 72279 | p1 | (T)11 |
| 31 | 22 | 76133 | 76154 | p2 | (AG)11 |
| 32 | 10 | 77714 | 77723 | p1 | (C)10 |
| 33 | 10 | 80667 | 80676 | p1 | (A)10 |
| 34 | 12 | 89117 | 89128 | p3 | (TAG)4 |
| 35 | 12 | 89553 | 89564 | p4 | (GGCG)3 |
| 36 | 12 | 91469 | 91480 | p3 | (CTT)4 |
| 37 | 12 | 99162 | 99173 | p4 | (GGGA)3 |
| 38 | 10 | 99182 | 99191 | p2 | (GA)5 |
| 39 | 12 | 99383 | 99394 | p4 | (GGCG)3 |
| 40 | 12 | 101601 | 101612 | p4 | (GCTA)3 |
| 41 | 10 | 104328 | 104337 | p1 | (T)10 |
| 42 | 10 | 104460 | 104469 | p2 | (AT)5 |
| 43 | 12 | 105014 | 105025 | p1 | (T)12 |
| 44 | 12 | 107319 | 107330 | p1 | (T)12 |
| 45 | 10 | 108507 | 108516 | p2 | (CT)5 |
| 46 | 12 | 120691 | 120702 | p4 | (GCCG)3 |
| 47 | 10 | 122441 | 122450 | p2 | (TA)5 |
| 48 | 10 | 125552 | 125561 | p1 | (A)10 |
| 49 | 12 | 133469 | 133480 | p4 | (ATAA)3 |
| 50 | 10 | 138308 | 138317 | p1 | (A)10 |
| 51 | 12 | 148010 | 148021 | p4 | (AAAG)3 |
| 52 | 12 | 149931 | 149942 | p4 | (GCTT)3 |
| 53 | 12 | 150784 | 150795 | p4 | (AAAG)3 |
| 54 | 12 | 153560 | 153571 | p4 | (CTTT)3 |
| 55 | 12 | 161730 | 161741 | p4 | (TTAC)3 |
| 56 | 15 | 164437 | 164451 | p1 | (T)15 |
| 57 | 12 | 168947 | 168958 | p4 | (ATGG)3 |
| 58 | 10 | 171654 | 171663 | p2 | (CT)5 |
| 59 | 12 | 172597 | 172608 | p3 | (TAG)4 |
| 60 | 11 | 174445 | 174455 | p1 | (C)11 |
| 61 | 12 | 176644 | 176655 | p4 | (TTCT)3 |
| 62 | 10 | 180280 | 180289 | p2 | (TC)5 |
| 63 | 12 | 188213 | 188224 | p4 | (AATG)3 |
| 64 | 12 | 188674 | 188685 | p4 | (AAGA)3 |
| 65 | 12 | 191345 | 191356 | p3 | (TAT)4 |
| 66 | 12 | 197043 | 197054 | p4 | (CAGT)3 |
| 67 | 15 | 200879 | 200893 | p5 | (ATACG)3 |
| 68 | 12 | 204852 | 204863 | p4 | (ATGA)3 |
