# Supplementary material for: Assembly and comparative analysis of the complete mitochondrial genome of Isopyrum anemonoides (Ranunculaceae)
Source: PLoS One. 2023 Oct 5;18(10):e0286628. doi: 10.1371/journal.pone.0286628 (PMC10553351; doi:10.1371/journal.pone.0286628)
Supplement: S6 Table — (DOCX) [file pone.0286628.s006.docx]

**S6 Table. Pairwise dN/dS ratios in different mitochondrial genes of 3 Ranunculaceae plants**

| **Genes** | ***I. anemonoides* vs *A. kusnezoffii*** | ***I. anemonoides* vs A. maxima** | **A.kusnezoffii vs A. maxima** |
| --- | --- | --- | --- |
| atp1 | 0.000 | 0.178 | 0.178 |
| atp4 | 0.534 | 1.135 | 1.200 |
| atp6 | 0.451 | 1.122 | 0.824 |
| atp8 | 0.282 | 0.813 | 0.489 |
| atp9 | 0.136 | 0.119 | 0.167 |
| ccmB | 2.197 | 0.000 | 1.719 |
| ccmC | 0.581 | 0.807 | 0.000 |
| ccmFC | 0.948 | 0.892 | 0.958 |
| ccmFN | 0.648 | 0.392 | 0.069 |
| cox1 | 0.161 | 0.083 | 0.310 |
| cox2 | 1.261 | 0.784 | 1.594 |
| cox3 | 0.221 | 1.018 | 0.520 |
| cytb | 0.038 | 0.129 | 0.152 |
| matR | 0.924 | 1.819 | 0.960 |
| mttB | 0.788 | 0.614 | 0.825 |
| nad1 | 1.065 | 1.502 | 1.570 |
| nad2 | 0.000 | 0.235 | 0.550 |
| nad4 | 0.363 | 0.446 | 1.426 |
| nad4L | 0.000 | 0.070 | 0.097 |
| nad5 | 0.264 | 0.607 | 0.674 |
| nad6 | 0.000 | 0.411 | 0.402 |
| nad7 | 1.098 | 0.783 | 0.937 |
| nad9 | 0.207 | 0.269 | 0.137 |
| rpl5 | 1.698 | 1.204 | 1.294 |
| rpl10 | 0.223 | 0.279 | 0.000 |
| rpl16 | 0.000 | 0.964 | 0.964 |
| rps3 | 0.712 | 0.852 | 0.463 |
| rps4 | 0.706 | 0.874 | 0.823 |
| rps7 | 0.781 | 1.291 | 1.624 |
| rps12 | 0.495 | 0.495 | 0.000 |
| rps13 | 1.070 | 0.578 | 1.422 |
| sdh4 | 0.000 | 1.527 | 0.984 |
